# Screening of the Two *Pseudochloris wilhelmii* Strains (Adriatic SAG 55.87 and Mangrove SAG 1.80) Cultured Under a Wide Range of Conditions: Nitrogen Concentration, Nitrogen Source and Salinity

**DOI:** 10.3390/microorganisms14071444

**Published:** 2026-06-30

**Authors:** Luka Žilić, Lara Jurković, Ines Haberle, Sunčana Geček, Maria Blažina

**Affiliations:** Institute Ruđer Bošković, Bijenička Cesta 54, 10000 Zagreb, Croatia; lzilic@irb.hr (L.Ž.); ihaberle@irb.hr (I.H.); suncana@irb.hr (S.G.); mblazina@irb.hr (M.B.)

**Keywords:** *Pseudochloris wilhelmii*, growth rate, salinity, nitrogen source, ammonia, nitrate, microalgae, picoeukaryotes

## Abstract

The growth of two strains of *Pseudochloris wilhelmii*, Adriatic strain SAG 55.87 and Mangrove strain SAG 1.80, is compared under different salinities, nitrogen sources and concentrations by a fast 96-well screening method. Cultures were grown in BG11 medium with modifications of nitrogen source (ammonium and nitrate) and nitrogen concentrations (0.3 to 19.2 mM) across salinities ranging from 2 to 24 PSU. Growth was measured by optical density at 690 nm, and specific growth rates were analyzed using a Bayesian generalized additive mixed model to identify optimal conditions. Both strains tolerate a broad range of salinities, but cell density was generally higher with nitrate as a nitrogen source than with ammonium nitrogen. The Adriatic strain showed better growth performance when grown on nitrate, with a mean observed growth rate of 0.143 d^−1^ and a 1.96-fold increase in OD_690_. The Mangrove strain showed narrower response and lower mean growth rates on nitrate (0.104 d^−1^). Overall, both strains showed similar growth performance on ammonium, with comparable mean growth rates of 0.0304 for the Adriatic strain and 0.0358 for the Mangrove strain. The results show high intraspecific variation in tolerance to salinity and nitrogen for *P. wilhelmii*. This 96-well screening method for microalgae cultivation is a powerful and rapid tool for narrowing down optimal growth conditions, as well as to be used as a guide for a larger-scale setup.

## 1. Introduction

One of the causes of global issues is anthropogenic pressures such as greenhouse gas emissions, environmental pollution, food stock problems, and unavailability of medicines. To mitigate all of these, microalgae can offer a solution through the potential to produce biomass for applications in various industries. Microalgae are microscopic, single-cell or multi-cellular photosynthetic organisms found either in freshwater or marine environments [1,2]. Microalgae have capabilities of generating highly valuable macromolecules like lipids, proteins and carbohydrates in larger amounts compared to terrestrial crops, which makes them more suitable for further research and development [1]. These macromolecules can be used to produce biofuels, food supplements and pharmaceuticals [3]. Beyond energy applications, food stocks and pharmaceuticals, biomass from microalgae provides a sustainable raw material for bioplastic production, forming biopolymers such as polyhydroxyalkanoates, with their biomass serving as a functional filler in composite materials [4]. Microalgae can also be used in integrated processes with industrial or municipal wastewaters and can reduce cultivation costs and close nutrient cycles [5,6]. This type of concept is called a circular bioeconomy, in which microalgae convert waste into valuable products and thereby mitigate eutrophication and greenhouse gas emissions. According to patent documents, the most frequently researched species are *Spirulina* and *Chlorella*, which are mainly the subject of previous cultivation research, commercial cultivation and application [7]. Despite the potential of microalgae to produce valuable products, industrial application is still constrained by strain-specific performance and the difficulty of identifying proper robust strains that are suited for particular biotechnological purposes.

Recent advances in the field of genetic engineering have opened new possibilities of modified strains that can produce higher contents of lipids, proteins and carbohydrates [5]. Even though it is highly unlikely to get as high concentrations of valuable compounds in a wild strain as in a genetically modified strain, there are substantially less concerns in cultivation of non-modified indigenous species strains. Large-scale production of wild autochthonous microalgal strains lowers the risk of environmental hazards linked to the release of genetically modified microorganisms into the environment and simultaneously avoids legal constraints related to their use.

Cultivation of marine microalgae provides certain advantages in comparison to the freshwater species, as they do not take any agricultural land or freshwater resources, thus avoiding food vs. fuel concerns [4]. Given their high potential, the use of microalgae for biomass production can be further improved, and development is on the rise.

Microalgae are also studied for their production of valuable metabolic products, such as lipids, carotenoids, carbohydrates, proteins and extracellular polysaccharides, which can be used for food, feed, nutraceuticals, pharmaceuticals, and biorefinery applications [8,9]. Accumulation of valuable metabolic products can be stimulated through biotechnological processes such as nutrient limitation, salinity stress, light and temperature regulation [9,10].

Marine microalgae, like the species *Pseudochloris wilhelmii*, are promising candidates for research due to their fast growth and wide nutrient (type and concentration) and salinity tolerance [11]. High tolerance levels are particularly relevant for biotechnological applications where organisms can be exposed to extreme, fluctuating, or suboptimal conditions, as well as for observing growth patterns within different strains [12].

Major environmental factors are the availability of nutrients, such as nitrogen and phosphorus, and salinity, which highly affect the physiology and productivity of marine microalgae. Nitrogen sources commonly used are nitrate (NO_3_^−^), ammonium (NH_4_^+^) and urea, with the latter two having potential toxic effects, affecting the assimilation process and possibly even causing physiological stress [13]. Recent studies show that nitrogen and phosphorus limitation strongly influence growth rates, production of metabolic products like lipids, proteins and carbohydrates, and the efficiency of photosynthesis in marine microalgae, with species-specific responses to different stress regimes [14,15,16].

Environmental conditions, for instance temperature, pH and nitrogen availability, have a strong influence on the stability and productivity of microalgal cultures [17]. Although temperature, pH and nitrogen are critical for productivity, overall balance between carbon, nitrogen and phosphorus determines the efficiency of cell growth while still maintaining high nutrient uptake [18].

For this reason, optimization of medium is crucial when selecting strains for biotechnological applications as in those intended for biomass production or wastewater treatment. Wastewater has a high concentration of ammonium nitrogen. Elevated concentrations of NH_4_^+^ partially convert to ammonia (NH_3_) depending on pH, temperature and salinity. Ammonia is more membrane-permeable and can inhibit growth or, at some concentrations, even become toxic [13]. Furthermore, *P. wilhelmii*, compared to extensively studied species such as *N. gaditana*, *Synechoccous* sp., *Chlorella* spp., and *Tetradesmus* spp., shows very promising performance in its removal rate of nitrogen, phosphorus and CO_2_ fixation, whilst reducing toxicity in challenging industrial effluents and achieving higher biomass under comparable conditions [6,11]. Salinity, in turn, affects osmotic balance, ion homeostasis, and nutrient uptake and interacts with nitrogen form to determine the overall physiological response of marine microalgae [3].

The recent literature shows an increase in usage of high-throughput screening (HTS) as a modern tool that has significantly improved microalgae strain selection and culture optimization [19,20,21,22]. Small assays conducted in microplates allow rapid evaluation of different strains and cultivation conditions in parallel [21]. HTS tests have shown that they can be used as an effective tool for assessing nutrient responses. In particular, HTS tests can show that nitrogen source, phosphorus availability, trace element balance and their interactions can strongly affect growth, the efficiency of photosynthesis, pH stability and biomass production [21,22].

Therefore, HTS is an efficient method for selecting strains suited to specific applications and for evaluating intra-specific variation in nutrient and salinity tolerance, making it a crucial step in development of microalgal processes for large-scale biomass and lipid production. In a paper published by Huerlimann, de Nys and Heimann, various species of tropical marine microalgae were grown in different standard media, showing that strain and species strongly influenced both biomass production and lipid content [23].

In our study, the two strains (SAG 55.87 and SAG 1.80) of the same species, *Pseudochloris wilhelmii*, were investigated. The strain *P. wilhelmii* SAG 55.87 was originally isolated from the Adriatic Sea, sampled near Rovinj (Croatia), while *P. wilhelmii* SAG 1.80 is associated with Mangrove swamp [24]. The aim of the study was to compare growth rates between the two strains across a range of salinities and nitrogen (as nutrient) concentrations given two different nitrogen sources, NO_3_^−^ and NH_4_^+^. This was done by utilizing a rapid screening, 96-well microplate method [25], using the optical density as a proxy for microalgal growth, and offering a fast and cost-effective way to test multiple strains’ growth across multiple screening conditions. By comparing these two strains in chosen conditions, we provide a new insight into intra-specific variation in nutrient and salinity tolerance in *P. wilhelmii* to optimize cultivation for biotechnological applications.

The strain *P. wilhelmii* SAG 55.87 was not investigated systematically before; therefore, this study is filling the knowledge gap mandatory for input data to cultivate this specific Adriatic strain in bioreactors or even on a higher scale.

## 2. Materials and Methods

### 2.1. Culture Conditions

The two *Pseudochloris wilhelmii* strains (Adriatic SAG 55.87 and Mangrove SAG 1.80) were provided by the Culture Collection of Algae at the University of Göttingen (SAG), Germany. The medium used for cell growth was modified Blue-Green medium (BG11 medium) [26] prepared by diluting artificial seawater (ASW) with double-distilled water to obtain salinities of 2, 4, 6, 8, 10, 12, 14, 16, 18, 20, 22 and 24 PSU.

BG11 medium was used as a base medium, differing only in nitrogen source. The first medium was an ammonium-based source of nitrogen that consisted of ammonium chloride (NH_4_Cl) and was modified to maintain the concentrations of 0.3, 0.6, 1.2, 2.4, 4.8, 9.6, and 19.2 mM. For the medium with the nitrate-based source of nitrogen that consisted of sodium nitrate (NaNO_3_), the concentrations of nitrate were kept the same as the ammonium one. In both cases, the phosphorus concentration was adjusted to Redfield ratio (N:P = 16:1). Nitrogen concentrations used for this experiment were chosen as a broad screening range to obtain strain responses under nitrogen-limited and nitrogen-rich conditions which were found previously to be significant for real wastewater [11,27].

Additional set of experiments was performed by replacing ASW in BG-11 medium with aged seawater. Aged seawater was prepared by placing the natural seawater in the dark for several months to deplete autotrophic organisms, which subsequently led to a decline in heterotrophic organisms due to the absence of their autotrophic food source. The aged seawater was filtered through 0.22 μm pore filter with the Millipore system.

### 2.2. Experiments

The effect of salinity and initial concentration of nitrogen on two *P. wilhelmii* strains (SAG 55.87 and SAG 1.80), for both ammonium (NH_4_^+^) and nitrate (NO_3_^−^) experimental setups, was observed during a period of 18 days. The cultures were grown in 96-well microplates in a climate chamber (Memmert ICH110L, software version AtmoCONTROL 2.11.0.0; Memmert GmbH + Co. KG, Schwabach, Germany) under controlled conditions at 24 °C, with a 12:12 h light:dark photoperiod and a photon flux density of 75 μmol photons m^−2^ s^−1^. The microplates were prepared by adding 270 µL of medium and 30 µL of inoculum into each well, while blank wells contained 300 µL of medium only. The plate layout was organized so that each row corresponded to a specific concentration, with row A containing the lowest concentration (0.3 mM) and row H containing the highest concentration (19.2 mM). Salinity was assigned across the columns, with column 1 corresponding to 2 PSU and column 12 to 24 PSU. The proxy for cell counts and cellular biomass was monitored by optical density at λ = 690 nm (OD_690_) and measured by Tecan Infinite M200 Pro multifunctional microplate reader and spectrophotometer (Tecan Group AG, Männedorf, Switzerland) with Magellan software (version 7.2). Each treatment was prepared in triplicate, and an additional plate served as a blank control containing sterile medium without inoculum. Furthermore, the blank control included all nitrogen concentrations and salinity levels corresponding to those applied in the triplicate treatment. To reduce potential bias caused by spatial variation within the climate chamber, randomization of samples was done by replacing the position of the plates. One replicate from the series with the strain 55.87 and NO_3_^−^ nitrogen source was excluded from the data analysis due to a technical error: the microplate dried out during the experiment, leading to unreliable measurements. Even though air flow is important to ensure uniform temperature, this highlights the importance of avoiding positioning plates near air-flow sources in future experiments using this method.

### 2.3. Analysis

The specific growth rate of cultivated strains (μ) was calculated for each day of the first week using a standard equation for intrinsic rate of increase [28] using OD_690_ measurements:

(1) $$\mu =\frac{ln\;\left({OD}_{t}\right)\;-ln\;\left({OD}_{t-1}\right)\;}{\Delta t}$$

where OD_t_ is the optical density at that day, OD_t−1_ at the day before, and Δt indicates time interval, i.e., 1 day. Mean growth rate for the exponential growth phase of the experiment was then calculated by averaging the specific growth rates of the first 11 days. Mean exponential-phase growth rates of the two tested strains and their responses across salinity and nitrogen concentration gradients were analyzed for each strain × nitrogen-source group (total of n = 924 observations) using a Bayesian generalized additive mixed model (Bayes GAMM) with a Student-t error distribution (Supplementary Information 2.1). This approach was selected because residual diagnostics from the initial Gaussian GAMM indicated deviations from normality and heavy-tailed residuals; the estimated median degrees of freedom (ν) of 2.75 (95% CI: 2.20–3.51) confirmed the necessity of this robust approach for handling outliers and non-normal error structures. The model included separate nonlinear response surfaces via tensor-product smooths of salinity and log_2_ nitrogen concentration for each strain × nitrogen-source group, while accounting for variation among the 11 experimental plates through random intercept. Analyses were implemented in the R package brms [19] using four Markov chain Monte Carlo (MCMC) chains with 4000 iterations each, including 2000 warm-up iterations. Convergence and sampling were evaluated using $\hat{R}$ values, effective sample sizes, posterior predictive checks, inspection for divergent transitions and tree-depth diagnostics, and trace plots, all of which indicated satisfactory model performance. The model was then used to predict optimal growth conditions for both strains and both nitrogen sources. The same Bayes GAMM approach was also used to analyze the log fold change in OD, i.e., ln(OD_final_/OD_init_), for the overall experiment period (18 days; results presented in Supplementary Information 2.2). Full details of the Bayesian GAMM methodology, model validation, and robustness analyses are provided in Supplementary Information 2 (Figure S1–S36 and Table S1–S14).

## 3. Results

### 3.1. Comparison of Adriatic and Mangrove Strain

**Figure 1:**
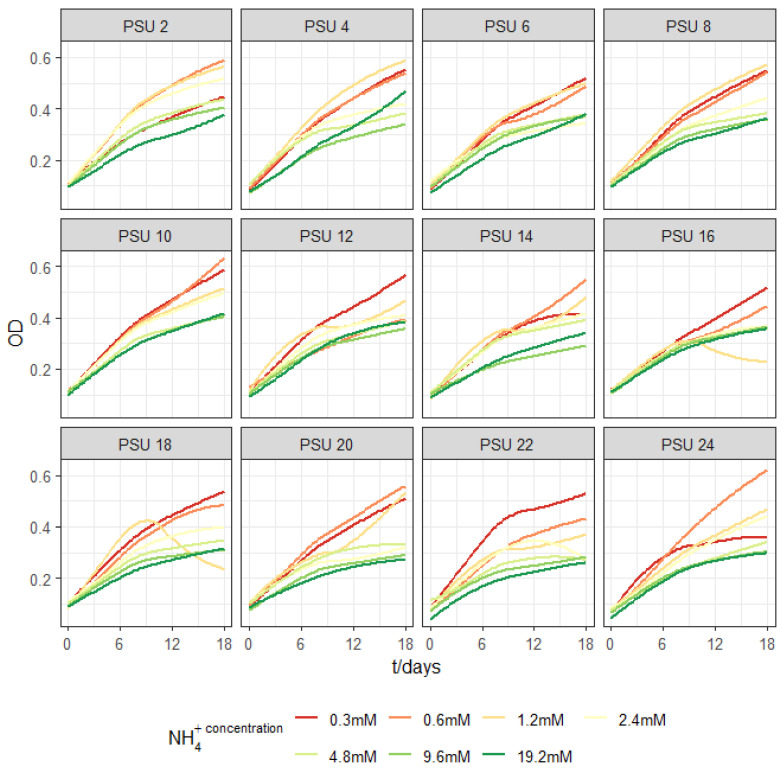
Growth as a function of OD_690_ measurement representing cell number of Adriatic strain (*P. wilhelmii* 55.87) at different initial ammonium nitrogen (NH_4_^+^) concentrations (shown in legend) and salinities (the title of each graph).

**Figure 2:**
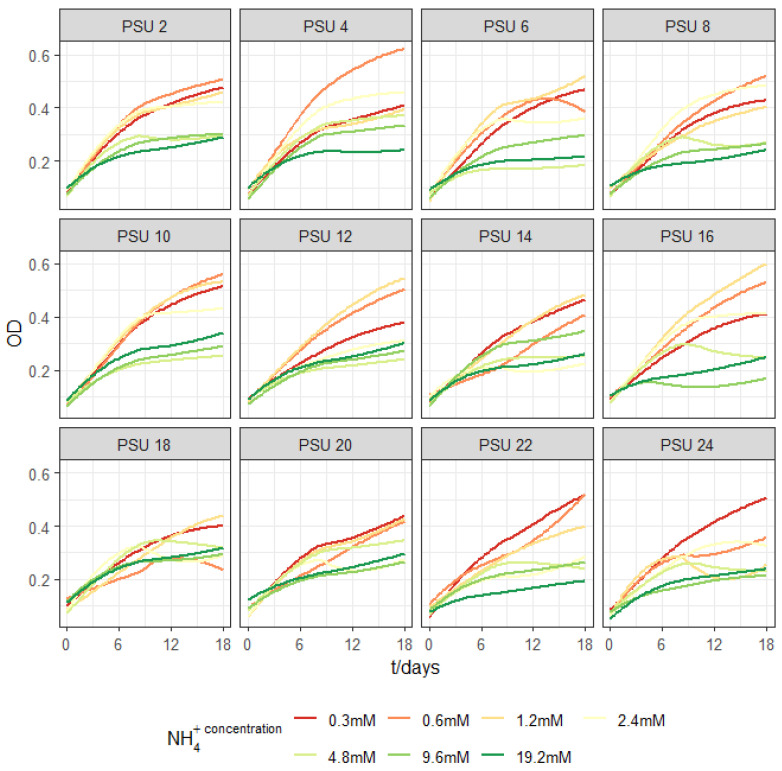
Growth as a function of OD_690_ measurement representing cell number of Mangrove strain (*P. wilhelmii* 1.80) at different initial ammonium nitrogen (NH_4_^+^) concentrations (shown in legend) and salinities (the title of each graph).

The two strains of *Pseudochloris wilhelmii* (Adriatic SAG 55.87 and Mangrove SAG 1.80) showed considerable cell density increase in the range of tested salinity and ammonium nitrogen (NH_4_^+^) concentration, as shown in Figure 1 and Figure 2, expressed as a function of measured OD_690_ in time, respectively. The measured data are represented as the day-to-day mean of replicate value lines. Cell density of the Adriatic strain had increased from approximately 0.1 to about 0.5 under most conditions and reached a maximum value of 0.6 at a concentration of 0.6 mM. If the 0.4 proxy formula is used for estimated biomass based on general green microalgal averages, the biomass productivity is 8.8 × 10^−3^ g L^−1^ day^−1^. In all salinities, the Adriatic strain (Figure 1) had higher cell densities on lower NH_4_^+^ concentrations. The smallest difference between the final OD_690_ among ammonium treatments is observed at 6 PSU, while the most distinct changes in growth under the different ammonium nitrogen concentrations were observed at 24 PSU. The calibration curve relating cell number to OD_690_ was done at ammonium nitrogen concentration of 0.6 mM and 19 PSU (Supplementary Information 1), giving the maximum cell number of 9.47 × 10^7^ mL^−1^. In the case of the Mangrove strain, by day 18, cell density had increased from approximately 0.1 to about 0.45, reaching a maximum value of 0.6 at concentrations of 0.6 and 1.2 mM at salinities 4 and 16 PSU, respectively. The estimated biomass ··productivity is 7.8 × 10^−3^ g L^−1^ day^−1^. To validate that absorbance reliably reflects biomass, the Mangrove strain (Figure 2) showed higher growth at lower NH_4_^+^ concentrations, with a comparable response across all salinity levels. The estimated biomass productivity is higher for the Adriatic strain by 1.0 × 10^−3^ g L^−1^ day.

**Figure 3:**
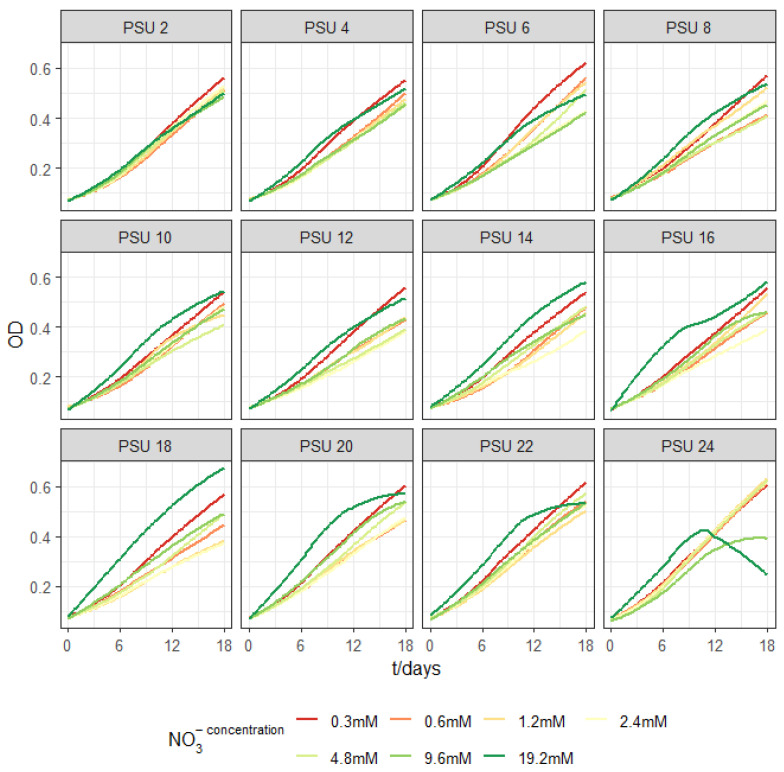
Growth as a function of OD_690_ measurement representing cell number of Adriatic strain (*P. wilhelmii* 55.87) at different initial nitrate (NO_3_^−^) concentrations (shown in legend) and salinities (the title of each graph).

**Figure 4:**
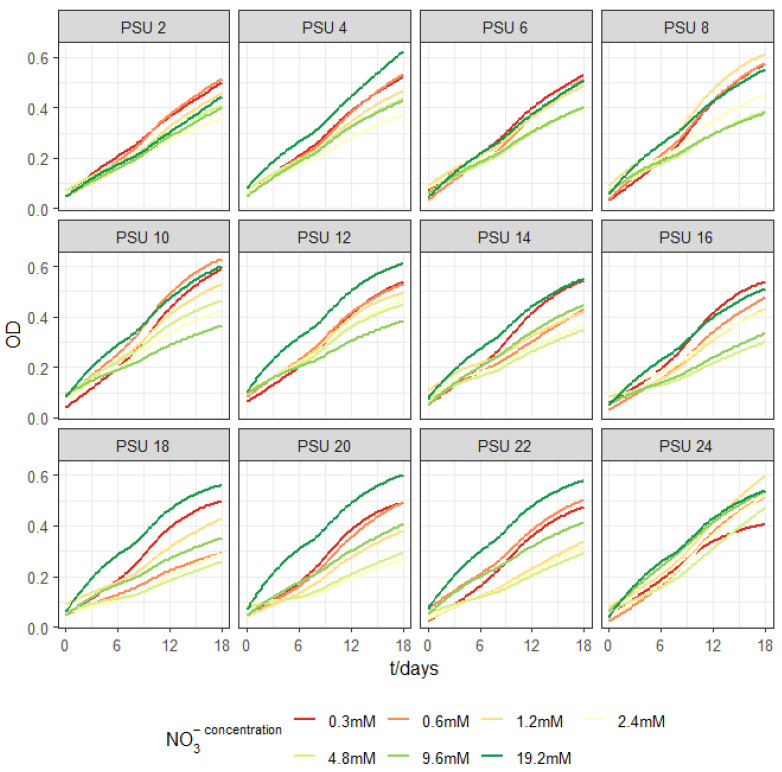
Growth as a function of OD_690_ measurement representing cell number of Mangrove strain (*P. wilhelmii* 1.80) at different initial nitrate (NO_3_^−^) concentrations (shown in legend) and salinities (the title of each graph).

The cell density of the Adriatic and Mangrove strains at different nitrate concentrations is shown at Figure 3 and Figure 4, respectively. Both strains showed considerable growth in a wide range of nitrate concentrations, but the relative performance of the Adriatic and Mangrove strains varied with salinity and nitrate concentration. In several conditions, the Adriatic strain reached OD_690_ values comparable to or higher than those of the Mangrove strain, with maximum cell density around 0.65, indicating that the medium with nitrate-based nitrogen supports strain-dependent growth rather than uniformly supporting growth across both strains. Mostly, OD_690_ values showed an increase in cell density from starting values of around 0.1 to maximum values of around 0.65 depending on the salinity and nitrogen concentration. The estimated biomass productivity is 1.2 × 10^−2^ g L^−1^ day^−1^, indicating higher biomass productivity with nitrate as a nitrogen source than ammonium nitrogen. The highest cell densities for Adriatic strain were obtained in higher salinities (PSU 18) and the highest nitrogen concentrations (19.2 mM) with maximum readings around 0.65, while the Mangrove strain had the maximum reading of around 0.6 in lower salinity (PSU 4) and at higher nitrogen concentrations (19.2 mM). Overall, cell density shows that both salinity and nitrate concentration strongly influence the growth dynamics of both strains.

### 3.2. Comparison of Nitrogen Intake Between NH_4_^+^ and NO_3_^−^ Source

The distinct difference between ammonium (NH_4_^+^) and nitrate (NO_3_^−^) as a source of nitrogen is that addition of NH_4_^+^ in culture media dissociates to ammonia NH_3_ which is balanced by pH, temperature and salinity. To compare the different nitrogen sources (NH_4_^+^ vs. NO_3_^−^), the experiments were performed in the same range of salinity (S = 2–24).

When comparing nitrogen sources within each strain, NO_3_^−^ generally indicates higher cell density development than NH_4_^+^ across the 18-day growth period. In the experiments conducted with NO_3_^−^, initial values were approximately 0.1, with final values increasing to about 0.60–0.65 by the day 18 at 19.2 mM-and-higher salinities (PSU 18–24), as shown in Figure 1 and Figure 2, with an overall increase of approximately 0.55. The estimated biomass productivity is 1.2 × 10^−2^ g L^−1^ day^−1^.

The experiments performed with NH_4_^+^, shown in Figure 3 and Figure 4, started at approximately 0.1 and increased to around 0.45–0.50 by day 18, which represents an overall change in OD of 0.40. The estimated biomass productivity is 8.8 × 10^−3^ g L^−1^ day^−1^. These results were obtained at the lower concentrations of NH_4_^+^ (0.6–1.2 mM^)^, with no substantial salinity effect. The differences between estimated biomass productivity in ammonium and nitrate-based medium is 3.2 × 10^−3^ g L^−1^ day^−1^, with higher mass productivity in nitrate medium.

If the experiments with the Adriatic strain with two nitrogen sources are compared, the differences between growth at different nitrate concentrations (Figure 3) are less pronounced at all salinities compared to growth with ammonium nitrogen (Figure 1). The reason for this is that ammonia (from speciation of NH_4_^+^) may inhibit growth; therefore, a reduction in growth with an increase in concentration is shown. In the case of different nitrate concentrations, the inhibition is not observed because the Adriatic strain takes the nitrogen in the amount that it needs to grow without restrictions. This may mean that the Adriatic strain is at its optimal condition.

However, the Mangrove strain (Figure 4) also shows small differences in growth under the different nitrate concentrations compared to the influence of ammonium nitrogen concentrations. But the difference in growth under nitrate concentrations is observed at higher salinity. The trends are not dependent on concentration, as it can be seen that a lower cell density is observed at a medium concentration of 4.8 mM. This strain is less adaptive to nutrient availability. Even though this is the same species, differences in where they are collected can change the adaptiveness of the species.

### 3.3. Growth Responses at Different Nitrogen Sources (Ammonium Nitrogen and Nitrate)

**Figure 5:**
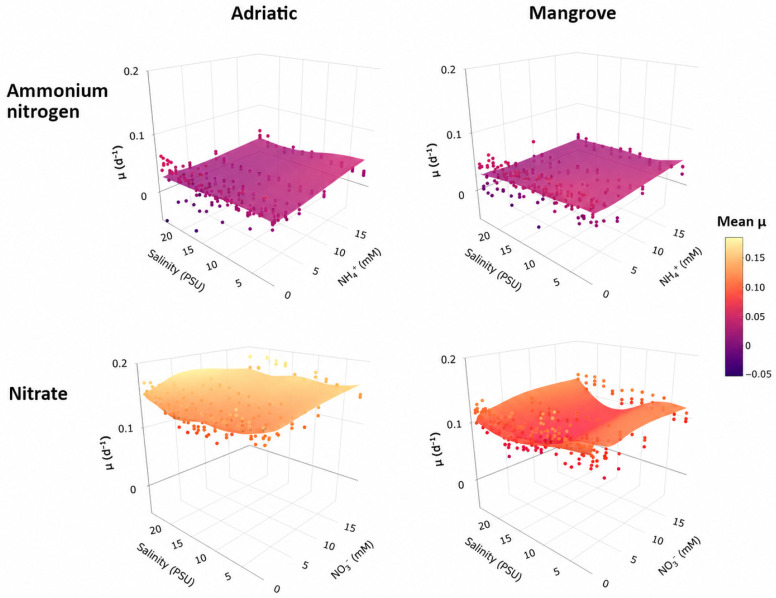
Visualization of growth rates based on OD_690_ across range of nitrogen concentrations and salinities of Adriatic (SAG 55.87 (**left**)) and Mangrove *P. wilhelmii* strain (SAG 1.80 (**right**)) with two different nitrogen sources (**top** vs. **bottom**). Dots are statistical mean value of measurements; surfaces are fits of the Bayesian general additive mixed model.

The specific growth rates were calculated as the average daily change in OD_690_ by Equation (1). The 3D visualization of growth rates for triplicates (two replicates for Adriatic-NH_4_^+^ as described before) is shown in Figure 5 as function of salinity and concentrations of nitrogen source (ammonium nitrogen (NH_4_^+^) or nitrate (NO_3_^−^)). The contours are a general additive model fitted to the mean measured growth rate values of the first weeks of each experiment, where the dots represent measured data. The first 11 days are shown due to the importance of the growth rate during the exponential phase for industrial use in remediation. A significant difference is observed between different sources, where growth is enormously faster in mediums with NO_3_^−^ than in those with NH_4_^+^ in both strains. The Adriatic strain grows significantly faster than the Mangrove strain with NO_3_^−^ as its nitrogen source.

To test the quality of aged seawater, the same experiments with NH_4_^+^ addition were done by replacing the artificial seawater (ASW) with filtered seawater which was kept in a dark place for few months (natural aged seawater, NASW). The cultures grew with a similar speed using the different waters (ASW and NASW).

**Figure 6:**
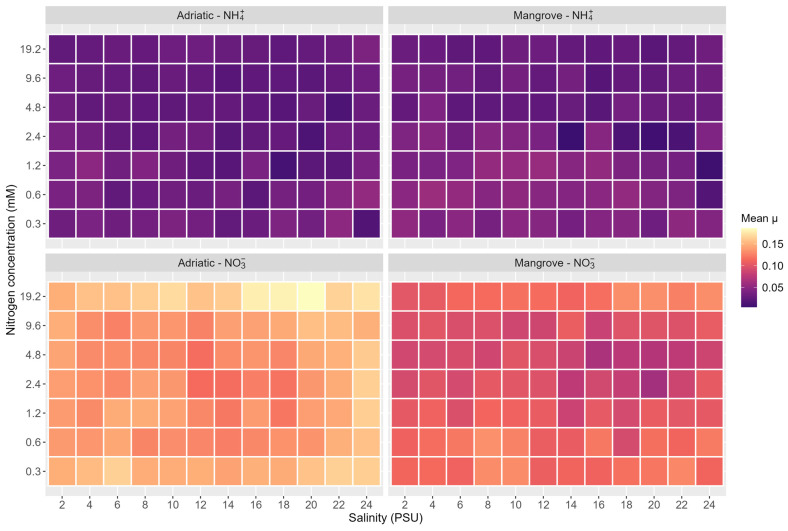
Heatmaps showing the mean observed growth rate (μ) across salinity and nitrogen concentration treatments for each strain and nitrogen source combination. Tile colors represent the mean growth rate calculated from plate replicates. Note that this is a discrete grid of treatment values.

Mean observed growth rates (μ) varied substantially among experimental groups, with the Adriatic strain showing both lowest (0.0304 day^−1^ for NH_4_^+^) and highest (0.1434 day^−1^ for NO_3_^−^) mean observed values (Figure 6, left top and bottom, respectively).

Applied to the observed measurements, the Bayes GAMM model convergence and fit were satisfactory: all parameters achieved $\hat{R}$ values ≤ 1.002, and effective sample sizes were substantial (e.g., intercept ESS bulk = 1600, and ESS tail = 2113), ensuring stable and well-sampled posterior distributions. Furthermore, there were zero divergent transitions during the sampling process, confirming the numerical stability of the final model. The predictions of the growth rates across tested scenarios, as well as predicted optimum regions, can be considered reliable.

**Figure 7:**
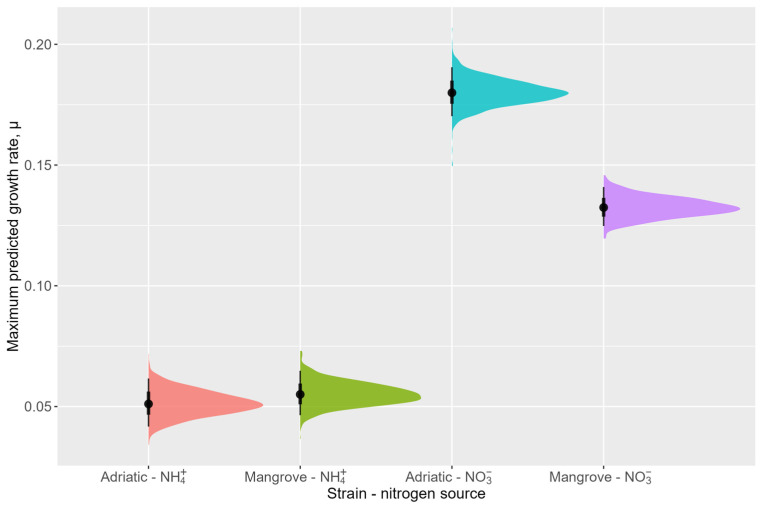
Posterior distribution of maximum predicted growth rate for each strain and corresponding nitrogen source based on estimates obtained from Bayesian GAMM model after combining the observed data and prior assumptions. The black points and error bars denote the posterior medians and 95% credible intervals, respectively.

**Figure 8:**
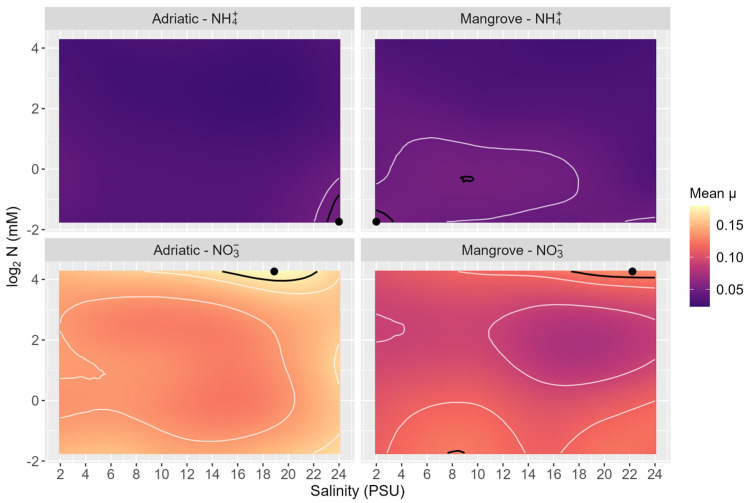
Posterior median response surfaces for growth rate (μ) across salinity and nitrogen concentration gradients for each experimental group, estimated from Bayesian GAMM model combining observed data with prior assumptions. Heatmaps show model predictions with plate-level random effects excluded, while white contour lines indicate gradients in predicted growth responses. White contour lines represent gradients in predicted growth responses, and black contour lines delineate the 95% optimal threshold regions. Note that nitrogen concentration is displayed on a log2-transformed scale to allow continuous visualization.

For both strains, an ammonium nitrogen source generally results in lower growth overall, reaching maximum predicted median growth rates of only 0.051 day^−1^ and 0.055 day^−1^ for the Adriatic and Mangrove strain, respectively (Figure 7; Table S6; 95% CI 5587: 0.042–0.062, CI 180: 0.046–0.065). In these treatments, performance is particularly reduced at intermediate to high salinities and nitrogen concentrations. The 95% optimum region of the Adriatic strain is very narrow, restricted to 45 grid points at high salinity and low NH_4_^+^ concentrations (Figure 8, top left; Table 1). The Mangrove strain has a comparable size of its acceptable growth region (41 grid points; Table 1) occurring at lower salinity and low NH_4_^+^ concentrations, with reduction in growth as salinity increases (Figure 8, top right). Overall, for the ammonium treatment, the maximum values are restricted to very low nitrogen concentrations for both strains—up to 0.82 and 0.54 mM for the Mangrove and Adriatic strain, respectively.

In contrast, the growth rate under the nitrate nitrogen source was consistently higher and showed smoother gradients and more uniformly elevated growth across a broad range of conditions. This was true especially for the Adriatic strain, which showed the highest and most clearly localized optima, i.e., a predicted median growth rate of 0.1795 day^−1^ (Figure 7, 95% CI: 0.1691–0.1904; Figure 8, bottom left), with an optimum region covering 130 grid points (Table 1). Regardless of such localized optima, this strain maintains a relatively high growth-rate values across a wide salinity range from 15 to 22 PSU (Table 1). The Mangrove strain has a slightly lower (100 grid points, Table 1) and less prominent optimum region. Interestingly, the optimum region spans wider salinities (8 to 24 PSU) compared to the Adriatic strain; however, this occurs with much lower optima (0.1308 d^−1^).

Pairwise comparisons confirmed that growth was significantly greater in nitrate-based media, with median differences in maximum μ of 0.1630 for the Adriatic strain and 0.0774 for the Mangrove strain compared to their ammonium counterparts (100% probability of superiority, Figure 7).

### 3.4. Long-Term Culture Performance and Biomass Accumulation

The main Bayesian GAMM analysis focused on the mean daily specific growth rate calculated over the first 11 days of the experiment, when most cultures were still in the active growth phase. This response variable provides a direct and biologically interpretable estimate of growth-rate responses to salinity and nitrogen availability, while minimizing the influence of later-stage processes such as nutrient depletion, density-dependent growth limitation, physiological acclimation, or the onset of stationary phase.

As a complementary endpoint analysis, longer-term net culture performance was evaluated over the full 18-day incubation period. This secondary analysis used the log-transformed ratio between final optical density on day 18 and initial optical density on day 0, thereby summarizing cumulative biomass accumulation across the entire experiment. This endpoint model was used to assess whether the treatment patterns identified during the active-growth window were retained in final biomass production.

The 18-day endpoint analysis (Supplementary Information 2) retained the main biological patterns identified by the 11-day growth-rate model. Nitrate remained the superior nitrogen source for both strains, and the Adriatic strain under nitrate showed the strongest predicted response in both analyses. In contrast, both ammonium treatments remained weak, with no clear difference between strains. Although the exact single-point optima did not match perfectly between the two models, except for the Adriatic strain under ammonium, the broader 95% near-optimum regions in the endpoint analysis encompassed the main optimum zones identified from the 11-day growth-rate analysis. Taken together, the endpoint analysis supports the conclusion that nitrate, particularly for the Adriatic strain, promotes the strongest and most sustained culture performance, whereas ammonium results in consistently low growth and limited final biomass accumulation.

## 4. Discussion

Microalgae from the genus *Pseudochloris* are characterized by rapid growth and tolerance to a wide range of nutrient concentrations and salinity, which makes them well suited to high-density biomass production and wastewater bioremediation. These screening experiments are a necessary first step for growth parameter optimization in a scaled-up bioprocess and further model simulation in photobioreactors [17,29]. The purpose of the scalability experiments is that they are used for accessing lipid fatty acid profiles to achieve better efficiency in microalgae energy harvesting [24,30].

For biotechnological productivity of microalgae, the mixture of seawater and wastewater is often used. Microalgae grown in seawater–wastewater mixtures effectively remove nitrogen and phosphorus while maintaining lipid production [4]. The performance depends on nutrient availability and CO_2_ conditions. Kinetic models were developed that show the importance of microalgae cultivation for wastewater treatment and carbon dioxide sequestration [31].

In this study, the two strains of *P. wilhelmii* are compared. The Adriatic strain *P. wilhelmii* SAG 55.87 was isolated near Rovinj at the RV001 station [30]. The conditions in Adriatic seawater are characterized by high salinity (around 38 PSU) and relatively warm temperature (10–24 °C) due to the shallow coastline. The Mangrove strain is *P. wilhelmii* SAG 1.80, which was originally isolated from Mangrove swamps in Bermuda, North America, and it is a freshwater species. Mangrove swamps are coastal wetlands found in tropical and subtropical regions and are characterized by salt-tolerant trees, shrubs, and plants growing in brackish or saline tidal waters [32]. The Mangrove strain needed to be adaptive to a high range of salinity changes. Therefore, the Adriatic strain is from seawater, and the Mangrove strain is a freshwater species but one that is found in a rapidly changing environment. By looking at Figure 5, it can be concluded that our growth data analysis shows that the Adriatic strain is more adaptive to salinity with nitrate as a nitrogen source than the Mangrove strain due to it reaching a higher specific growth rate in the tested range. Even if the species is the same, the place of isolation makes a big difference in adaptation. This is a good trait for using the Adriatic strain in industrial cultivation for biomass production. This can be compared to research in Turkey, where authors investigated the microalgae from different water resources for biotechnological utilization [33].

The optimal growth parameters are at the maximum 3D surface (Figure 5). The Adriatic strain exhibits the highest nitrogen concentrations with nitrate as the nitrogen source and at a salinity of 20 PSU. The Mangrove strain demonstrated its highest growth-rate values across a broader salinity range (8–24 PSU), which is consistent with its ecological origin, whereas the Adriatic strain showed a more pronounced optimum under marine salinity conditions. Nitrate is shown to be a better nitrogen source than ammonium nitrogen, as can be seen in Figure 5, with significantly lower mean specific growth (0.03 per day) while the specific growth with a nitrate source is in the range of 0.100 to 0.187 per day for the Adriatic strain and in the range from 0.046 to 0.152 per day for the Mangrove strain (Table S1). This is opposite to what is found in the literature on *Desmodesmus* sp. and *Scenedesmus obliquus*, where the authors stated that ammonium is the most preferred nitrogen source because its assimilation requires less energy [31]. The authors included ammonium speciation in the kinetic modeling of microalgae for wastewater treatment. The ammonium exists in equilibrium with ammonia depending on pH, with increasing free ammonia concentration with culture pH value. In our study the initial pH was 8; we calculated, by the MINTEQ program, the concentration of ammonia to be 0.01, 0.02, 0.04, 0.08, 0.16, 0.32 and 0.65 at 19 PSU, respectively, to ammonium concentrations added in experiments. The faster growth in nitrate medium can be explained with the toxic high concentration of free ammonia in our experimental conditions, which was also previously observed in the literature on the marine chlorophyte *Tetraselmis* sp. [34]. The study on *Tetraselmis* sp. showed more biomass achieved under nitrate (1.45 g/L) than ammonia (0.98 g/L). There was also more lipid produced from nitrate (19.9 mg/L day) as a source than ammonium nitrogen (14.4 mg/L day). There are also other species which prefer nitrate over ammonium for growth, such as *Botryococcus braunii* [35] and *Dunaliella tertiolecta* [36]. However, in the literature, some species, such as *Microchloropsis* and *Tetraselmis* [37], grow partly independent of external nitrate levels, suggesting possible internal nutrient storage and decoupled uptake dynamics.

In a study on the joint effect of ammonium and pH on the growth of *Chlorella vulgaris*, a significant reduction in specific growth was observed at the free ammonia concentration of 0.29 mM and pH of 7.5 while at pH 8.5, the reduction was at a free ammonia concentration of 0.59 mM [38]. This is in good correlation with our experiments where pH was in the middle of those two pH values (8.0) and the free ammonia concentration varied between 0.01 and 0.65 mM.

Based on Bayesian growth-rate analysis, the inferred optimum regions suggest that nitrogen form, rather than nitrogen availability alone, was the major driver of *P. wilhelmii* performance. In both strains, ammonium optima occurred at the lowest tested NH_4_^+^ concentrations, indicating that higher ammonium availability did not stimulate growth but instead became unfavorable. This is biologically plausible because ammonium, although energetically inexpensive to assimilate, can inhibit microalgal growth at elevated concentrations through ammonium or free-ammonia toxicity and associated physiological stress. Conversely, nitrate optima occurred at high nitrate concentrations, suggesting that nitrate was not inhibitory within the tested range and provided a more suitable nitrogen source for sustained growth. The location of nitrate optima near the upper end of the experimental gradient further suggests that nitrate saturation may not have been fully reached.

The salinity component of the optimum regions indicates that both strains performed best under moderate-to-high salinity conditions, particularly in nitrate media. This is consistent with a saline-adapted or halotolerant growth strategy, in which photosynthesis, osmotic regulation, and nutrient assimilation remain effective at elevated salinity. Differences in the width of the near-optimum regions further suggest strain-specific ecological strategies: broader regions indicate tolerance across a wider salinity range, whereas narrower high-performance regions indicate more specialized conditions for maximal growth. Taken together, the optimum regions indicate that nitrate combined with moderate-to-high salinity promotes the strongest growth response, while ammonium restricts growth to a narrow low-concentration range.

Utilization of biomass quantity and quality is driven by light exposure, temperature and nutrient availability, especially of nitrogen, which is a very abundant nutrient in wastewater. For example [39], *Pseudoneochloris marina* show best growth at ~28 °C, high light intesity, and high nitrogen concentrations; this can even cause stress conditions and shift its composition toward higher protein but lower lipids/carotenoids. This agrees with our results that the best growth is at the highest concentration of nitrate.

Our group’s previous work was done on different microalgae species but also on Northen Adriatic cyanobacteria [5,40]. The ammonium concentration was tested in the range of 0.8–3.2 mM in different salinities (0–35 PSU) to analyze the lipid, carbohydrate and protein dynamics, showing the importance of screening in microwells to obtain the optimal conditions for photobioreactors.

In other research, the microalgae *Pseudochloris wilhelmii* (Adriatic strain SAG 55.87), *Nannochloropsis gaditana* and prokaryote *Synechococcus* sp. MK568070 were examined for cultivation potential in oil refinery wastewater [6]. The highest specific nitrogen uptake rate was observed in *P. wilhelmii* (0.895 mmol/(gday) along with the highest volumetric productivity (93.9 mg/L/day) and wastewater toxicity removal (76.5%). The growth parameters were light intensity of 130 μmol photons m^−2^ s^−1^ with a light:dark photoperiod of 12:12 h and temperature of 24 °C, the same conditions as we use in this work. The medium was 1:1 artificial seawater and wastewater with salinity of 19 PSU and ammonium concentration of 1.2 mM. Salinity and ammonium concentrations were at similar values as in this paper. Our results shows that even though the productivity for the Adriatic strain was relatively high, it can be even better with these optimized parameters.

Futhermore, the Adriatic *P. wilhelmii* SAG 55.87 strain was also compared to *Microchloropsis gaditana* by cultivation in oil refinery wastewater [41]. There, *M. gaditana* accumulated up to 45% of total lipids when grown in optimal temperature and illumination. *M. gaditana* is one of the prominent microalgae species for biofuel production; the paper shows that *P. wilhelmii* SAG 55.87 did not differ considerably from *M. gaditana.* In the same paper, both species tolerate ammonium (NH_4_^+^/NH_3_) up to16 mM in the appropriate media at pH 8.3 and temperature 25 °C. Similar conditions (temperature of 24 °C and pH of 8.0) are noted in this paper, while the concentration of the ammonium nitrogen was up to 19.2 mM, and a good growing curve was also observed at all salinities (Figure 1). The *M. gaditana* showed slower growth in freshwater conditions (0 PSU), indicating marine nature, while Adriatic *P. wilhelmii* grew notably even at 0 PSU for 6 days. The Adriatic *P. wilhelmii* growth varies with salinity, with the fastest growth observed in the range from 7 PSU to 21 PSU, which is in good agreement with our findings. There were lower concentrations of NH_4_^+^ tested (1.2, 1.6, 2.0 and 2.4 mM), but it was from oil refinery wastewater which had all other contaminants.

Another strain of the class *Trebouxiophyceae*, *Picochlorum atomus* also showed growth across a wide salinity range with little effect on productivity [42]. The authors concluded that nutrient limitation (not salinity) is the main driver of increased lipid accumulation. In our study, growth is also affected by nitrogen concentration, while salinity did not exhibit a significant role on growth. Not only was wastewater treatment used, but rather, wastewater centrate can be used for production of microalgae [43]. Wastewater centrate can support *M. gaditana* growth at 30 °C in raceway reactors and tubular photobioreactors, but high ammonium inhibits productivity. The authors show that adjusting the N:P ratio improves biomass and nutrient removal. In comparison, our N:P ratio was constant at 16:1, and we also saw the negative influence of ammonium on specific growth rate compared to nitrate as a source.

The base for preparing the culture medium is aged seawater, but in the future, work will mostly be carried out under the radar. Nobody is speaking about it, but everybody is using it if they are doing fine and precise work. Aged seawater may be a cost-effective medium component for cultivating microalgae because it lowers the need for freshwater demand while preserving the ionic composition and trace elements of the natural seawater [11]. Aging in the dark can also reduce labile dissolved organic carbon and decrease inorganic nitrogen and phosphorus, thus providing a lower background medium for cultivation [11,44,45]. Here, we tested the difference between aged natural seawater and artificial seawater (Figure 5). The results obtained with artificial seawater were comparable to those of aged natural seawater, demonstrating that artificial seawater is a cost-effective alternative for microalgae cultivation that eliminates the need for preparation process and chemical usage.

The 96-well approach for the cultivation of microalgae was proposed by Nowark in 2005 [46]. Even before, the method was described by Podola and Melkonian in 2003 for operating an algal biosensor designed for the rapid detection of volatile toxic compounds, specifically methanol and formaldehyde [47]. However, little research has been done on this topic [25,48,49], which gives our research added value.

Specific growth rates of two microalgae species, *Scenedesmus* sp. UTEX1589 and an environmental isolate (Strain 1), were tested by a group in Mexico [48]. The system produces tightly clustered, reproducible data with a low coefficient of variation ranging from 6.8% to 8.3%. The broad statistical analyses were done, and statistical robustness showed that randomizing treatment placement within the internal wells keeps overall data variance (CV 9.4–12.9%) well below standard toxicity testing limits (20%). This study was more focused on high CO_2_ concentration but can used as a reference for the method that we used.

A group in Denmark tested *Nannochloropsis salina* and *Chlorella sorokiniana* specific growth rates by repeated batch culturing in microplates supplied with continuous light at six light intensities (up to 136 μmol photons m^−2^ s^−1^) [25]. The research was done with 24-well plates so the volume was bigger, and the optical density (OD) was measured at 750 nm; fluorescence was also measured at 440 nm emission: 690 nm detection. By changing the light intensity, they were able to apply the kinetic models; at the same time, the method is not as fast as that used in our research, and only one parameter was tested. In microplate cultures, fluorescence has a lower detection limit than optical density for a range of species, proving that our choice of analysis by OD_690_ was correct. A group from Iran has also done experiments in 24-well plates with *Picochlorum* sp. D8 isolated from the Persian Gulf [50]. The research focused on optimal conditions as a concentration of phosphate and iron(III) chloride hexahydrate in Rudic’s medium. The source of nitrogen was nitrate. The carbohydrate content was determined. Compared with our study, this research has significantly larger volume; therefore, additional analysis of metabolites was carried out. The optimized medium consisted of phosphate, 0.148 g/L, and iron(III) chloride hexahydrate, 0.009 g/L, which enhanced carbohydrate production by approximately 3.5-fold (2401.53 μg/mL) compared to what was obtained in the original Rudic’s medium (693.84 μg/mL). Two experimental designs were tested—Plackett–Burman Design and Central Composite Design—with a focus on carbohydrate content, while our study focused on obtaining the optimal conditions for scaling-up. Compared to our study, the design was done to be as close to bioreactor conditions as possible. However, the limitations of a 96-well microplate-based design need to be stated; they include evaporation due to low volume, known limitations associated with mass transfer, light distribution, and oxygen availability. The advantage is a significantly higher number of experiments, which we had 504 of, while in planned design, like Plackett–Burman design and central composite design, only 14 runs each were carried out.

Another study introduced a novel 96-well microplate-based assay for the culture, monitoring, and in vivo phenotypic screening of the model microalga *Chlamydomonas reinhardtii* [49]. The study broadened the use of this method to the toxicity assay of silver nanoparticles on the common *Chlamydomonas reinhardtii* wild-type strain CC-124, also known as 137c, and growth conditions varied throughout trials in terms of media, vessel type (flask or microplate), microplate shaker speed (flasks were grown at 100 rpm), and photoperiod. This also proves that the method used in this study provides a fast, low-cost, and low-waste alternative to traditional flask cultivation.

High throughput was also done in even smaller volumes, at 150 μL on *Chlamydomonas reinhardtii* and *Chlorella* sp., 11_H5 isolated in Australia, in the literature [51]. A light optimization screen is designed to identify species-specific illumination conditions that maximize photosynthetic efficiency and productivity to fast-track systems optimization. The results from this high-throughput screening were used in two more studies on modeling dynamics [19] and prediction of microalgae growth dynamics in photobioreactors [20]. This confirms that even with the limitation, this high-throughput method is reliable for screening the optimal conditions which can be applied to photobioreactors.

## 5. Conclusions

The fast screening of parameters for cultivation optimization of two *Pseudochloris wilhelmii* strains was done. The changing parameters were salinity (2–24 PSU) and nitrogen concentration (0.3–19.2 mM) in two forms—ammonium nitrogen and nitrate—while the temperature (24 °C), Redfield ratio (N:P = 16:1), light intensity (75 μmol photons m^−2^ s^−1^) and light cycle (12:12 h) were maintained constant.

The only difference in strains is the origin of isolation: the Adriatic SAG 55.87 strain and the Mangrove SAG 1.80 strain. The Adriatic strain grows significantly better than the Mangrove across the tested nitrate range, with no difference compared to the Mangrove in the ammonium nitrogen, indicating that it is a preferred strain for photobioreactors. This is based on initial screening, but for better validation, an experiment on a larger scale is necessary. The nitrate is a better nutrient source than ammonium nitrogen for both strains. The Mangrove strain is more adaptable to salinity change. Furthermore, the natural aged seawater shows comparable growth as medium prepared with ASW, thus suggesting a possible cost-effective alternative and a simplified alternative of preparing medium for algae cultivation.

This screening is important for getting faster results in scaled-up production like photobioreactors (PBRs) and even open raceway pond cultivation. The Adriatic *P. wilhelmii* is neglected but could be good match for starting algae cultivation on the Adriatic coast.

## Figures and Tables

**Table 1:** Point optima and 95% near-optimum regions for the growth rate (μ). Column n-grid represents the number of grid points within the tested salinity and nitrogen parameter space that fall into the 95% near-optimum region.

**Group**	**Salinity, PSU**		***c*** **(N), mM**		**Log_2_N, mM**		**Max Predicted** ** µ, d^−1^**	**n-Grid**
	**min**	**max**	**min**	**max**	**min**	**max**		
Adriatic NH_4_^+^	23.11	24.00	0.30	0.54	−1.74	−0.89	0.0508	45
Mangrove NH_4_^+^	2.00	9.56	0.30	0.82	−1.74	−0.28	0.0534	41
Adriatic NO_3_^−^	14.89	22.22	15.56	19.20	3.96	4.26	0.1795	130
Mangrove NO_3_^−^	7.78	24.00	0.3	19.20	−1.74	4.26	0.1308	100

## Data Availability

The original contributions presented in this study are included in the article/Supplementary Material. Further inquiries can be directed to the corresponding author.

## Supplementary Materials

The following supporting information can be downloaded at: https://www.mdpi.com/article/10.3390/microorganisms14071444/s1, Figure S1: Calibration curve of cell number per mL, counted on an optical microscope, as function of OD_690_, as a test for reliability; Figure S2: Distribution of $\hat{R}$ values across monitored parameters. Values close to 1 indicate agreement among MCMC chains; Figure S3: Trace plots for the residual scale *σ* and Student-*t* degrees-of-freedom parameter *ν*. Well-mixed chains without drift support stable posterior sampling; Figure S4: Posterior distribution of the Student-*t* degrees-of-freedom parameter. Low values indicate heavy-tailed residual variation; Figure S5: Posterior predictive density overlay for the marginal distribution of growth rates; Figure S6: Posterior predictive empirical cumulative distribution function overlay; Figure S7: Posterior predictive check for the overall mean; Figure S8: Posterior predictive check for the overall standard deviation; Figure S9: Group-specific posterior predictive density overlays; Figure S10: Residual-like values plotted against posterior mean fitted values, separately by group; Figure S11: Residual-like values by strain × nitrogen-source group; Figure S12: Residual-like values by experimental plate; Figure S13: Posterior estimates of plate-level random intercepts; Figure S14: Posterior median population-level response surfaces, excluding plate-level random effects; Figure S15: Prediction uncertainty surface, expressed as the width of the 95% credible interval; Figure S16: Predicted optima and 95% near-optimum regions. Contour indicates combinations with posterior median predicted growth at least 95% of the group-specific maximum; Figure S17: Posterior uncertainty in optimum location based on draw-wise maxima; Figure S18: Posterior distributions of maximum predicted growth by group; Figure S19: Observed mean log fold change in OD surface by group. This plot summarizes raw means before model fitting and provides a visual check that the modelled response surfaces reflect the empirical structure of the data; Figure S20: Distribution of $\hat{R}$ values across monitored parameters for the log fold change in OD model. Values close to 1 indicate agreement among MCMC chains; Figure S21: Trace plots for residual scale σ and Student-*t* degrees-of-freedom parameter ν in the log fold change in OD model. Well-mixed chains without drift support stable posterior sampling; Figure S22: Posterior distribution of the Student-*t* degrees-of-freedom parameter for the log fold change in OD model. Low values indicate heavy-tailed residual variation; Figure S23: Posterior predictive density overlay for the marginal distribution of log fold change in OD; Figure S24: Posterior predictive ECDF overlay for log fold change in OD; Figure S25: Posterior predictive check for the overall mean of log fold change in OD; Figure S26: Posterior predictive check for the overall standard deviation of log fold change in OD; Figure S27: Group-specific posterior predictive density overlays for log fold change in OD; Figure S28: Residual-like values plotted against posterior mean fitted values for log fold change in OD, separately by group; Figure S29: Residual-like values by strain × nitrogen-source group for log fold change in OD; Figure S30: Residual-like values by experimental plate for log fold change in OD; Figure S31: Posterior estimates of plate-level random intercepts for log fold change in OD; Figure S32: Posterior median population-level response surfaces for log fold change in OD, excluding plate-level random effects; Figure S33: Prediction uncertainty surface (95% credible interval width) for log fold change in OD; Figure S34: Predicted optima and 95% near-optimum regions for log fold change in OD. Contours indicate combinations with posterior median response at least 95% of the group-specific maximum; Figure S35: Posterior uncertainty in optimum location based on draw-wise maxima for log fold change in OD; Figure S36: Posterior distributions of the maximum predicted log fold change in OD for each experimental group; Table S1: Summary of the observed growth rate by strain × nitrogen-source group; Table S2: Prior distributions used in the Bayesian GAMM; Table S3: Summary of MCMC diagnostics and key posterior predictive check for the final Student-*t* Bayesian GAMM; Table S4: Posterior estimates of plate-level random intercepts. 5587: Adriatic strain, 180: Mangrove strain; Table S5: Point optima and 95% near-optimum regions. Point optima are defined as the maximum of the posterior median response surface. Near-optimum regions include grid points with predicted median growth ≥ 95% of the group-specific maximum. 5587: Adriatic strain, 180: Mangrove strain; Table S6: Posterior uncertainty in optimum location. Values are posterior medians with 95% credible intervals obtained from draw-wise maximum locations. 5587: Adriatic strain, 180: Mangrove strain; Table S7: Pairwise posterior differences in maximum predicted growth. Positive values mean that the first group in the contrast has a larger maximum than the second group. 5587: Adriatic strain, 180: Mangrove strain; Table S8: Summary of the observed log fold change in OD690 by strain × nitrogen-source group for log fold change in OD by group; Table S9: Prior distributions for the log fold change in OD Bayesian GAMM; Table S10: Summary of MCMC diagnostics for the log fold change in OD Student-*t* Bayesian GAMM; Table S11: Posterior estimates of plate-level random intercepts for log fold change in OD. 5587: Adriatic strain, 180: Mangrove strain; Table S12: Point optima and 95% near-optimum regions for predicted log fold change in OD. 5587: Adriatic strain, 180: Mangrove strain; Table S13: Values are posterior medians with 95% credible intervals obtained from draw-wise maximum locations. 5587: Adriatic strain, 180: Mangrove strain; Table S14: Pairwise posterior differences in maximum predicted log fold change in OD. Positive values indicate that the first group has a larger maximum than the second group; included in the Supplementary Materials [52,53,54].
