# Supplementary material for: Screening of the Two Pseudochloris wilhelmii Strains (Adriatic SAG 55.87 and Mangrove SAG 1.80) Cultured Under a Wide Range of Conditions: Nitrogen Concentration, Nitrogen Source and Salinity
Source: Microorganisms. 2026 Jun 30;14(7):1444. doi: 10.3390/microorganisms14071444 (PMC13414064; doi:10.3390/microorganisms14071444)
Supplement: Supplementary file 1 [file microorganisms-14-01444-s001.zip › microorganisms-4357912-supplementary 1.pdf]

Supplementary Information

Screening of the two *Pseudochloris wilhelmii* strains (Adriatic SAG 55.87 and Mangrove SAG 1.80) cultured under a wide range of conditions: Nitrogen concentration, nitrogen source and salinity

Luka Žilić, Lara Jurković*, Ines Haberle, Sunčana Geček, Maria Blažina

Ruđer Bošković Institute, Croatia

June 29, 2026

Supplementary information 1: Calibration curve

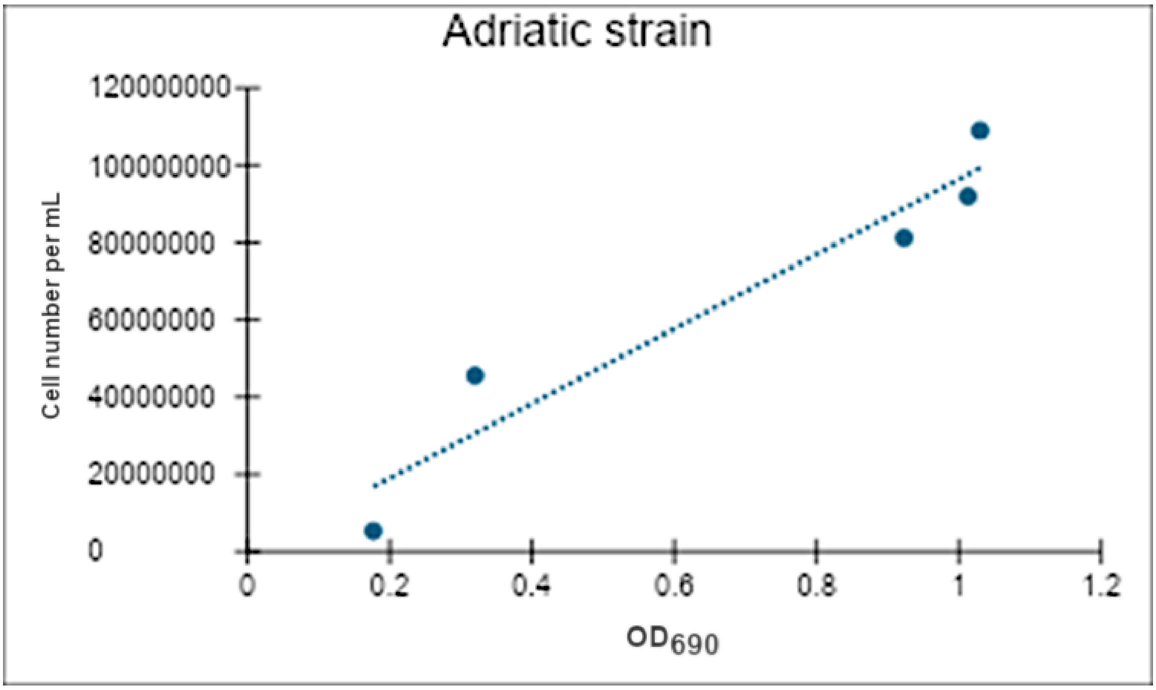

Figure S1: Calibration curve of cell number per mL, counted on an optical microscope, as function of OD_{690}, as a test for reliability.

Supplementary information 2: Supplementary Methods and Validation Report of Bayesian GAMM analysis of *P. wilhelmii* growth

This supplementary report documents the Bayesian generalized additive mixed models (Bayesian GAMMs) used to analyse growth rates and log fold changes in optical density (OD_{690}) of *P. wilhelmii* across salinity and nitrogen-concentration gradients. It is intended to provide the additional information regarding prior distributions, sensitivity/robustness checks, and model validation procedures.

1 SI2: Growth rate responses

1.1 Data structure and experimental design

The analysis included 924 observations distributed across four strain × nitrogen-source groups and 11 experimental plates. Nitrogen concentration was analysed on the $\log_2$ scale because the experimental concentration series followed a two-fold dilution design. Thus, one unit on the transformed scale corresponds to a doubling of nitrogen concentration.

Table S1: Summary of the observed growth rate by strain × nitrogen-source group.

Group	ID	n	Plates	Mean μ	SD μ	Min μ	Max μ
Adriatic - NH4+	5587_NH4	252	3	0.0304	0.0136	-0.0536	0.0644
Mangrove - NH4+	180_NH4	252	3	0.0358	0.0161	-0.0366	0.0681
Adriatic - NO3-	5587_NO3	168	2	0.1434	0.0168	0.1002	0.1872
Mangrove - NO3-	180_NO3	252	3	0.1035	0.0186	0.0463	0.1517

1.2 Bayesian GAMM specification

Let y_i denote the mean exponential-phase growth rate for observation i. The fitted model was

$$y_i \sim t_\nu(\mu_i, \sigma), \tag{S1}$$
$$\mu_i = \alpha_{g[i]} + f_{g[i]}(s_i, \log_2 N_i) + b_{p[i]}, \tag{S2}$$
$$b_p \sim \mathcal{N}(0, \sigma_{\text{plate}}), \tag{S3}$$

where $g[i]$ indexes the strain × nitrogen-source group, s_i is salinity, N_i is nitrogen concentration, and $p[i]$ indexes the experimental plate. The function $f_g(\cdot)$ is a group-specific tensor-product smooth, allowing each strain × nitrogen-source group to have a separate nonlinear salinity × nitrogen response surface.

The model was implemented in `brms` as

```
mu_val ~ group +
  t2(salinity, log2_N, by = group, k = c(6, 5)) +
  (1 | plate_ID)
family = student()
```

The Student-t likelihood was used because preliminary Gaussian GAMM diagnostics showed non-normal, heavy-tailed residuals, especially in the ammonium treatments. Student-t model directly estimates the degrees-of-freedom parameter ν, where smaller values indicate heavier residual tails and stronger robustness to outlying observations.

1.3 Prior distributions

The model used weakly informative priors chosen on the observed scale of growth rates. These priors regularize implausibly large effects while allowing the data to dominate the posterior estimates.

Table S2: Prior distributions used in the Bayesian GAMM.

Model component	Prior
Intercept	Student-$t(3, 0, 0.1)$
Population-level coefficients	$\mathcal{N}(0, 0.1)$
Residual scale σ	Exponential(20)
Plate-level SD σ_{plate}	Exponential(10)
Student-t df ν	Gamma$(2, 0.1)$

1.4 Posterior computation and MCMC diagnostics

The model was fitted using four Markov chain Monte Carlo chains, each with 4000 iterations, including 2000 warm-up iterations. This yielded 8000 post-warmup posterior draws. Sampling was performed with Hamiltonian Monte Carlo using the No-U-Turn Sampler. Convergence and sampling quality were assessed using $\hat{R}$, effective sample size (ESS), divergent transitions, and tree-depth diagnostics.

Table S3: Summary of MCMC diagnostics and key posterior predictive check for the final Student-t Bayesian GAMM.

Diagnostic	Value
Number of post-warmup draws	8000
Maximum $\hat{R}$ across monitored parameters	1.002
Minimum bulk ESS	1600
Minimum tail ESS	2113
Divergent transitions	0
Maximum observed tree depth	8
Transitions at tree depth ≥ 10	0
Student-t ν, posterior median [95% CrI]	2.75 [2.20, 3.51]
Observed mean vs posterior predictive mean	0.0723 vs 0.0747 [0.0722, 0.0773]

All monitored parameters had $\hat{R} \leq 1.002$, with minimum bulk ESS of 1600 and minimum tail ESS of 2113. There were no divergent transitions and the sampler did not reach problematic tree-depth saturation. These diagnostics indicate satisfactory convergence and stable posterior sampling.

The posterior median of the Student-t degrees-of-freedom parameter was $\nu = 2.75$ (95% CrI: 2.20–3.51), supporting the use of a heavy-tailed residual distribution rather than a Gaussian likelihood.

1.5 Model validation procedures

Model validation was based on posterior predictive checks, residual-like diagnostics, and inspection of plate-level random effects.

1.5.1 Posterior predictive checks

Posterior predictive checks compare the observed data with replicated datasets simulated from the fitted posterior predictive distribution. If the model is adequate, the observed data should look typical relative to the model-generated data.

The overall posterior predictive mean showed only a small discrepancy: the observed mean growth rate was 0.0723, whereas the posterior predictive median was 0.0747 (95% interval: 0.0722–0.0773). The absolute difference was 0.0024, corresponding to a small tendency to over-predict the overall mean, but not one considered biologically substantial.

The density, ECDF, mean, standard deviation, and grouped density posterior predictive checks are shown in Supplementary Figures S5–S10.

1.5.2 Residual-like checks

Although residuals are not the primary diagnostic in Bayesian posterior predictive workflows, residual-like values $y_i - \mathbb{E}(y_i \mid \text{posterior})$ were used to inspect systematic bias across fitted values, groups, and plates. These diagnostics are shown in Supplementary Figures S11–S13.

1.5.3 Plate-level variation

Plate-level random intercepts were minimal and centered around zero, showing no consistent directional bias related to nitrogen source (Figure S13). Observed shifts represent random experimental noise rather than systematic treatment effects, confirming that block-level variation was successfully controlled without dominating the primary biological signals.

Table S4: Posterior estimates of plate-level random intercepts. 5587: Adriatic strain, 180: Mangrove strain.

Plate ID	Estimate	95% CrI
NH4_5587_blok2	-0.0021	[-0.0082, 0.0038]
NH4_5587_blok3	-0.0005	[-0.0066, 0.0054]
NH4_5587_blok4	0.0022	[-0.0039, 0.0082]
NH4_180_blok2	0.0003	[-0.0059, 0.0064]
NH4_180_blok3	-0.0002	[-0.0063, 0.0059]
NH4_180_blok4	0.0001	[-0.0061, 0.0061]
NO3_5587_blok2	-0.0025	[-0.0099, 0.0048]
NO3_5587_blok3	0.0028	[-0.0045, 0.0103]
NO3_180_blok1	0.0056	[-0.0005, 0.0121]
NO3_180_blok2	0.0014	[-0.0047, 0.0077]
NO3_180_blok3	-0.0067	[-0.0131, -0.0007]

1.6 Robustness and sensitivity analyses

The reviewer requested information on sensitivity analyses. The following robustness checks were implemented or derived from the final analysis outputs.

1.6.1 Sensitivity to the Gaussian residual assumption

The final model used a Student-t residual distribution because initial Gaussian GAMM diagnostics indicated non-normality and heavy-tailed residuals. The fitted Student-t model estimated $nu = 2.75$ (95% CrI: 2.20–3.51), a low value indicating strong residual heavy-tailedness. This quantitatively supports the use of a robust likelihood.

1.6.2 Sensitivity to plate-level structure

The model included 11 plate-level random intercepts. The posterior random-effect estimates were small relative to the major differences among nitrogen-source groups. Importantly, the ammonium plates containing extreme observations did not show a large whole-plate displacement, suggesting that the heavy tails reflected individual observations rather than a systematic block-wide shift.

1.6.3 Sensitivity of optimum inference

Optimum estimation was not based on a single maximum point only. Three complementary summaries were used:

1. the maximum of the posterior median response surface;
2. the 95% near-optimum region, defined as all grid points with posterior median predicted growth at least 95% of the group-specific maximum;
3. the posterior distribution of optimum locations obtained by identifying the maximum grid point separately within each posterior draw.

This approach avoids overinterpreting a single grid point and directly reports the uncertainty in optimum location.

1.7 Predicted response surfaces and optimum regions

Population-level response surfaces were predicted with plate random effects excluded, so that the surfaces represent expected group-level responses rather than any specific plate. The posterior median response surfaces and uncertainty surfaces are shown in Supplementary Figures S14 and S15.

Table S5: Point optima and 95% near-optimum regions. Point optima are defined as the maximum of the posterior median response surface. Near-optimum regions include grid points with predicted median growth $\geq$ 95% of the group-specific maximum. 5587: Adriatic strain, 180: Mangrove strain.

Group	Opt. PSU	Opt. N	Max μ [95% CrI]	95% salinity	95% N range	Grid points
5587_NH4	24.0	0.3	0.0508 [0.042, 0.062]	[23.11, 24.0]	[0.3, 0.54]	45
180_NH4	2.0	0.3	0.0534 [0.046, 0.065]	[2.0, 9.56]	[0.3, 0.82]	41
5587_NO3	19.1	19.2	0.1795 [0.170, 0.191]	[14.89, 22.22]	[15.56, 19.2]	130
180_NO3	22.0	19.2	0.1308 [0.125, 0.141]	[7.78, 24.0]	[0.3, 19.2]	100

The strongest growth response occurred in nitrate media. The highest maximum predicted growth was estimated for the Adriatic strain in nitrate (5587_NO3; $\mu = 0.180$), followed by the Mangrove strain (180_NO3; $\mu = 0.132$), both exceeding the ammonium treatments. In contrast, both ammonium groups showed much lower maximum predicted growth rate, with optima occurring at the lowest ammonium concentration tested (0.3). he Mangrove strain showed broader conditions ranges in both nitrate and ammonium treatments, but a smaller overall near-optimal area compared to the Adriatic strain.

Table S6: Posterior uncertainty in optimum location. Values are posterior medians with 95% credible intervals obtained from draw-wise maximum locations. 5587: Adriatic strain, 180: Mangrove strain.

Group	Salinity (PSU)	N concentration (mM)	μ at optimum
5587_NH4	24.0 [24.0, 24.0]	0.3 [0.3, 0.7]	0.051 [0.042, 0.062]
180_NH4	2.0 [2.0, 24.0]	0.3 [0.3, 1.0]	0.055 [0.046, 0.065]
5587_NO3	19.1 [17.1, 24.0]	19.2 [19.2, 19.2]	0.180 [0.170, 0.191]
180_NO3	22.0 [8.0, 24.0]	19.2 [0.3, 19.2]	0.132 [0.125, 0.141]

For some groups, particularly Mangrove strain (180_NH4 and 180_NO3), the posterior optimum location was broad. This indicates a relatively flat near-optimal surface and supports interpreting these groups in terms of near-optimum regions rather than a single precise optimum.

1.8 Pairwise comparisons of maximum predicted growth

Pairwise comparisons were computed from posterior draws of the maximum predicted growth for each group. These comparisons summarize differences in group-specific maximum growth and should not be interpreted as pointwise comparisons across the entire response surface.

Table S7: Pairwise posterior differences in maximum predicted growth. Positive values mean that the first group in the contrast has a larger maximum than the second group. 5587: Adriatic strain, 180: Mangrove strain.

Contrast	Median difference	95% CrI	Pr(diff > 0)	Pr(diff < 0)
5587_NH4 - 5587_NO3	-0.1630	[-0.1786, -0.1466]	0.000	1.000
5587_NH4 - 180_NO3	-0.0817	[-0.0949, -0.0685]	0.000	1.000
180_NH4 - 5587_NH4	0.0039	[-0.0100, 0.0181]	0.712	0.288
180_NH4 - 5587_NO3	-0.1590	[-0.1742, -0.1431]	0.000	1.000
180_NO3 - 5587_NO3	-0.0820	[-0.0962, -0.0665]	0.000	1.000
180_NH4 - 180_NO3	-0.0774	[-0.0895, -0.0650]	0.000	1.000

The posterior comparisons showed a clear ranking of maximum predicted growth:

$$\texttt{5587\_NO3} > \texttt{180\_NO3} \gg \texttt{180\_NH4} \approx \texttt{5587\_NH4}.$$

Both nitrate groups had substantially higher maximum predicted growth than both ammonium groups, while the two ammonium groups did not differ clearly from each other.

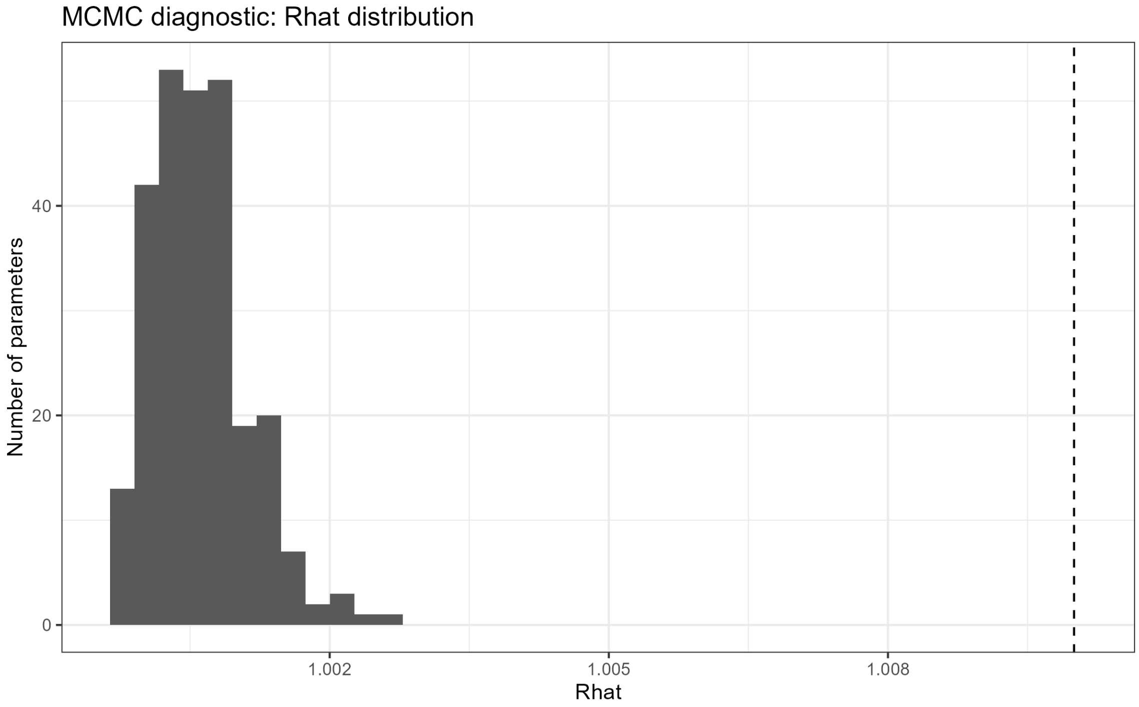

Figure S2: Distribution of $\hat{R}$ values across monitored parameters. Values close to 1 indicate agreement among MCMC chains.

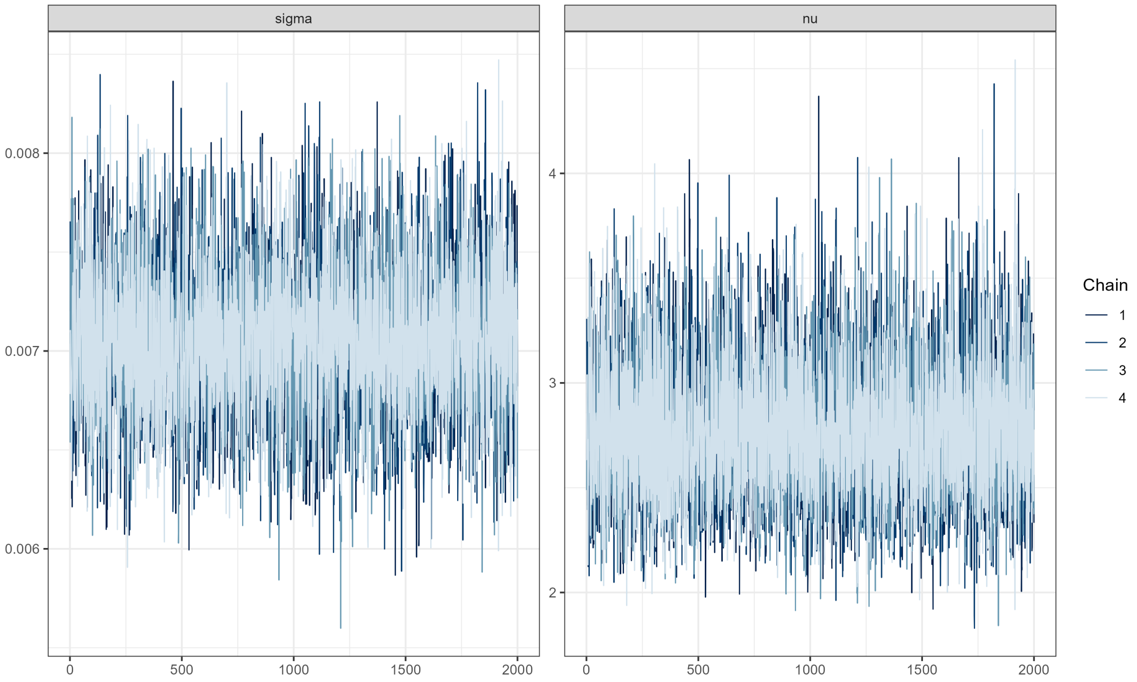

Figure S3: Trace plots for the residual scale σ and Student-t degrees-of-freedom parameter ν. Well-mixed chains without drift support stable posterior sampling.

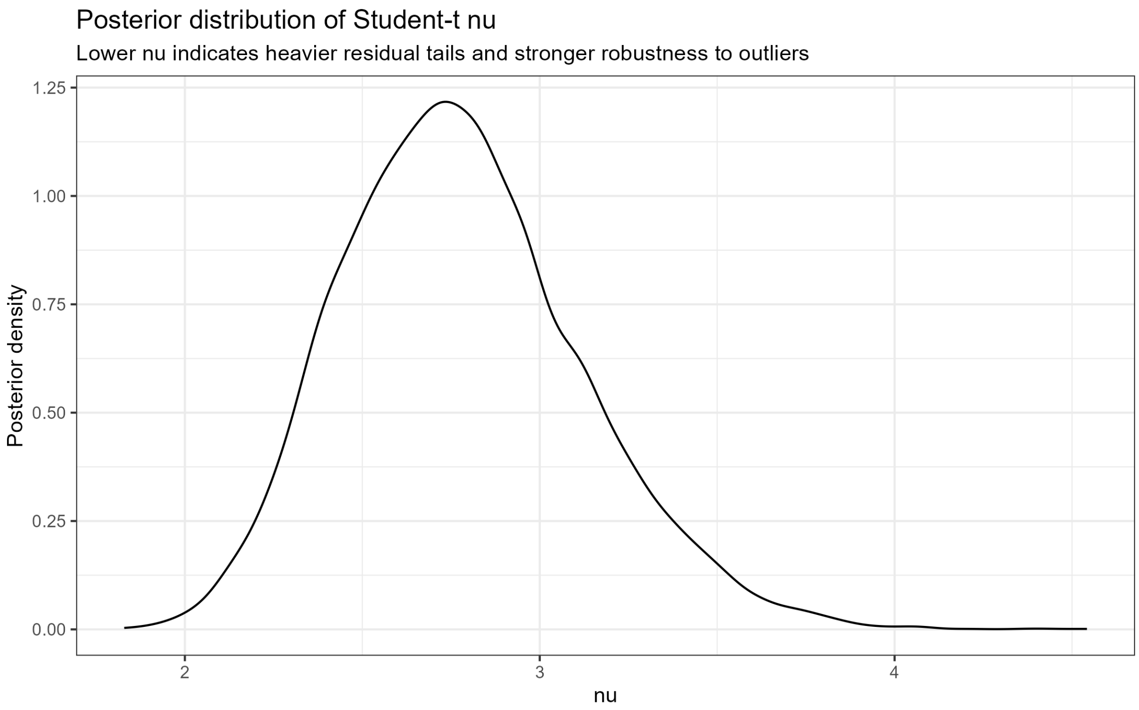

Figure S4: Posterior distribution of the Student-t degrees-of-freedom parameter. Low values indicate heavy-tailed residual variation.

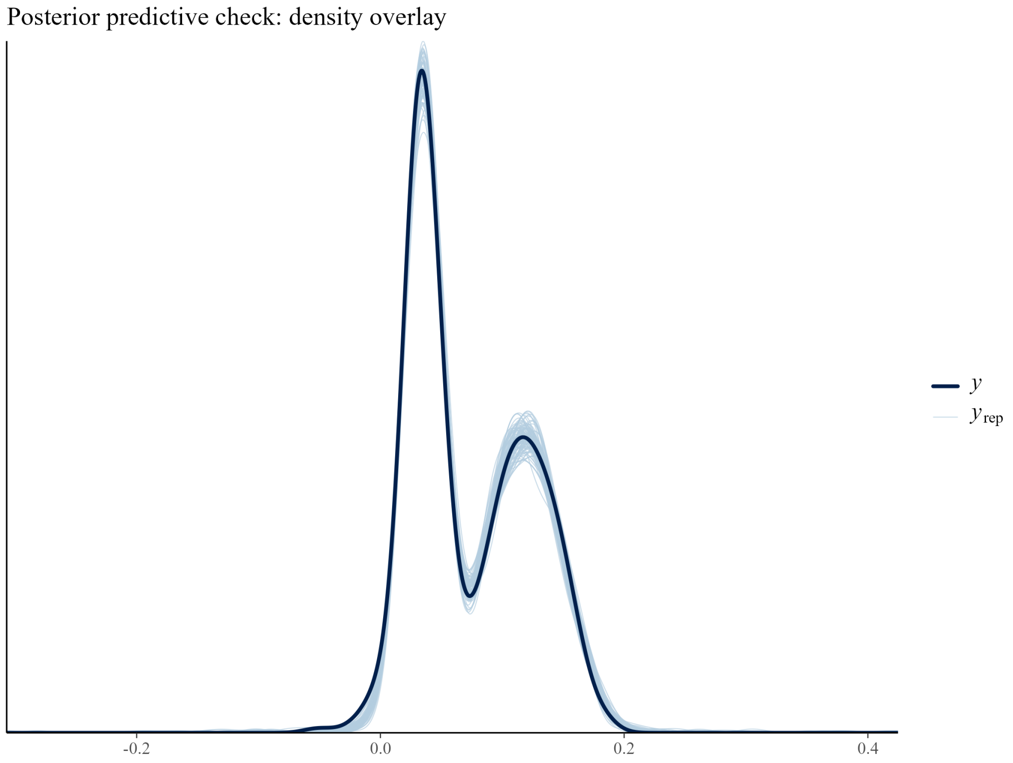

Figure S5: Posterior predictive density overlay for the marginal distribution of growth rates.

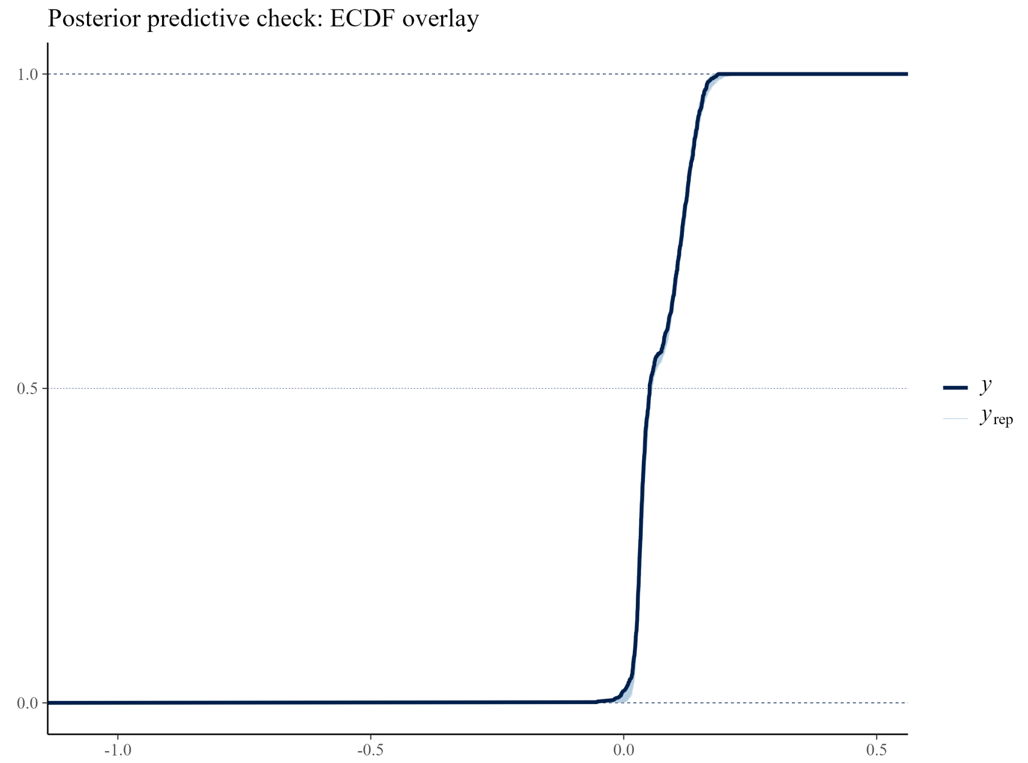

Figure S6: Posterior predictive empirical cumulative distribution function overlay.

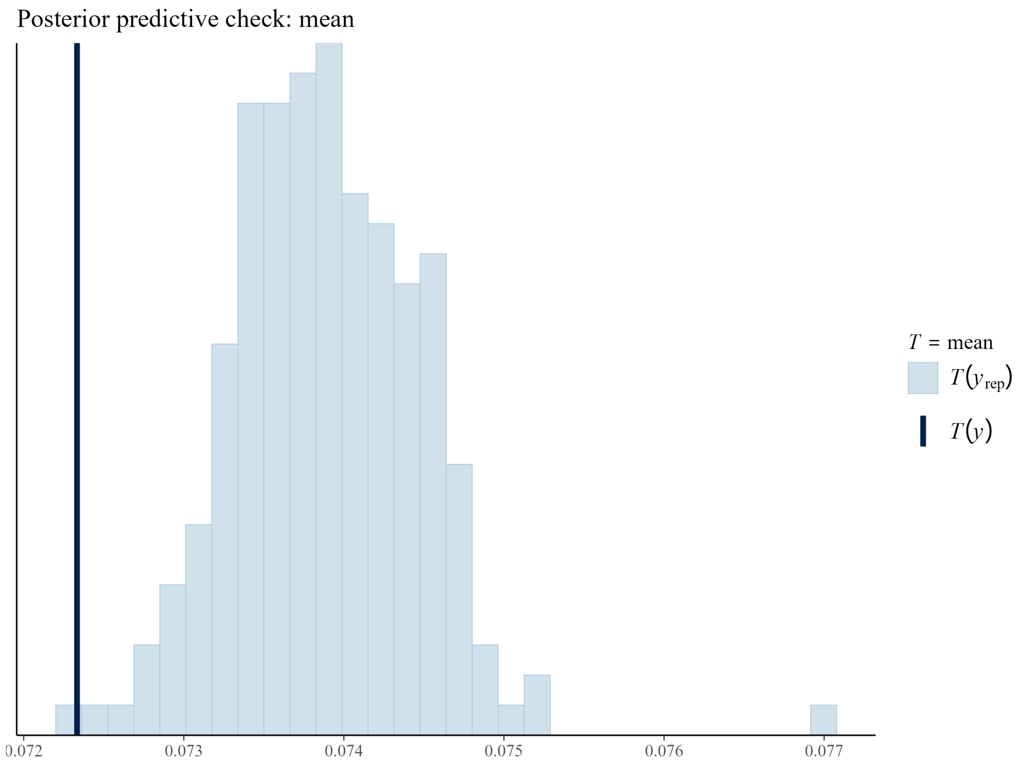

Figure S7: Posterior predictive check for the overall mean.

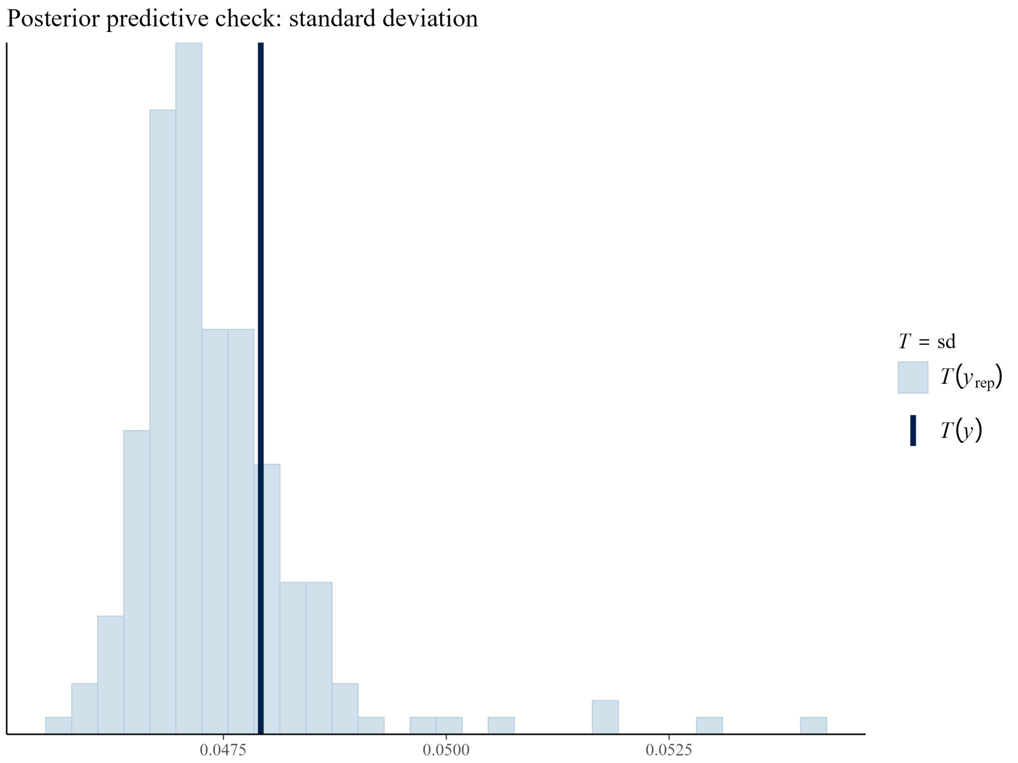

Figure S8: Posterior predictive check for the overall standard deviation.

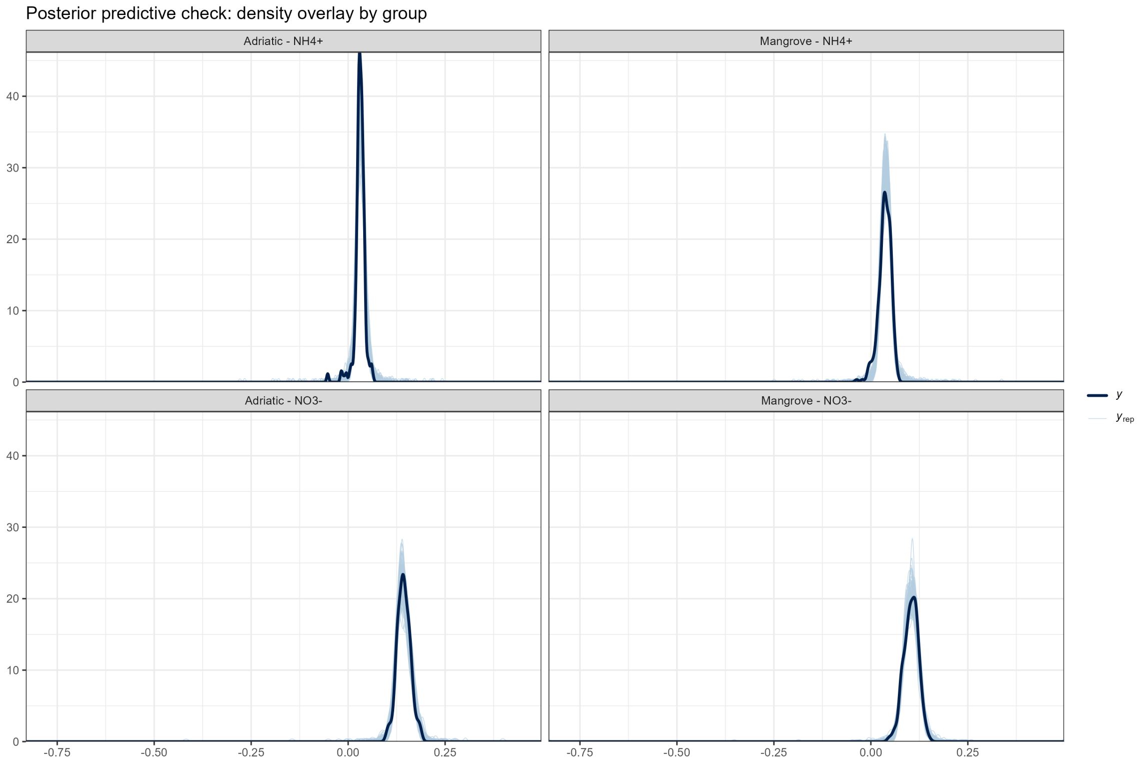

Figure S9: Group-specific posterior predictive density overlays.

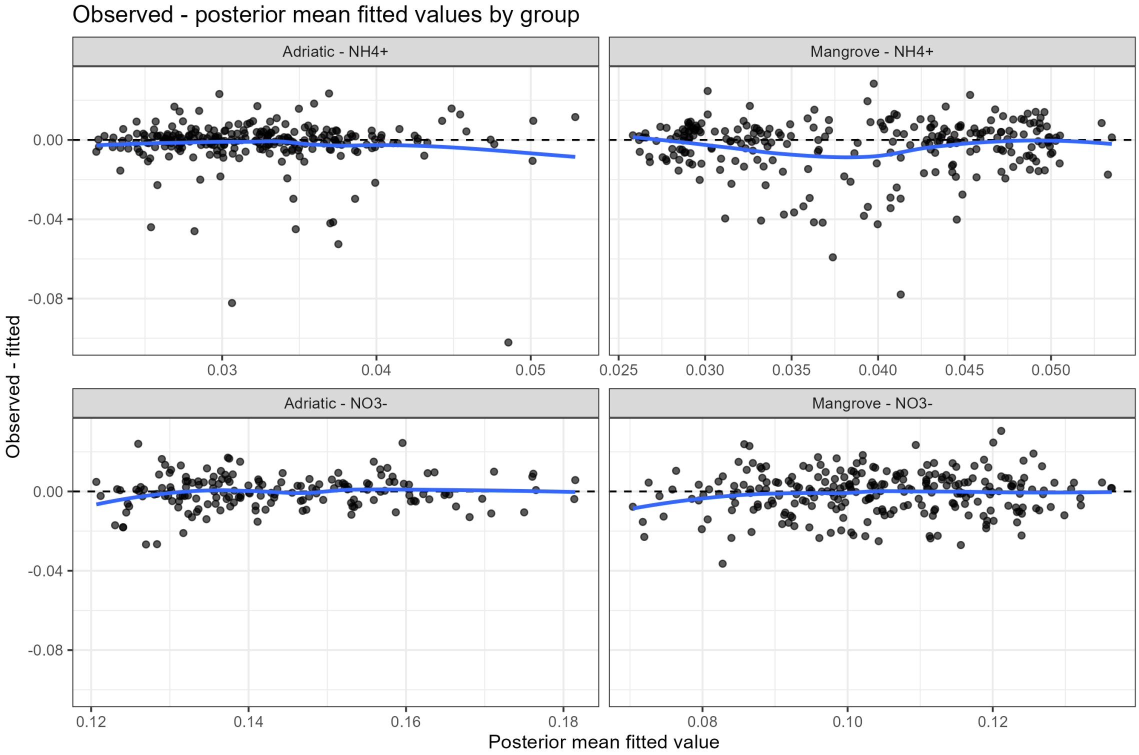

Figure S10: Residual-like values plotted against posterior mean fitted values, separately by group.

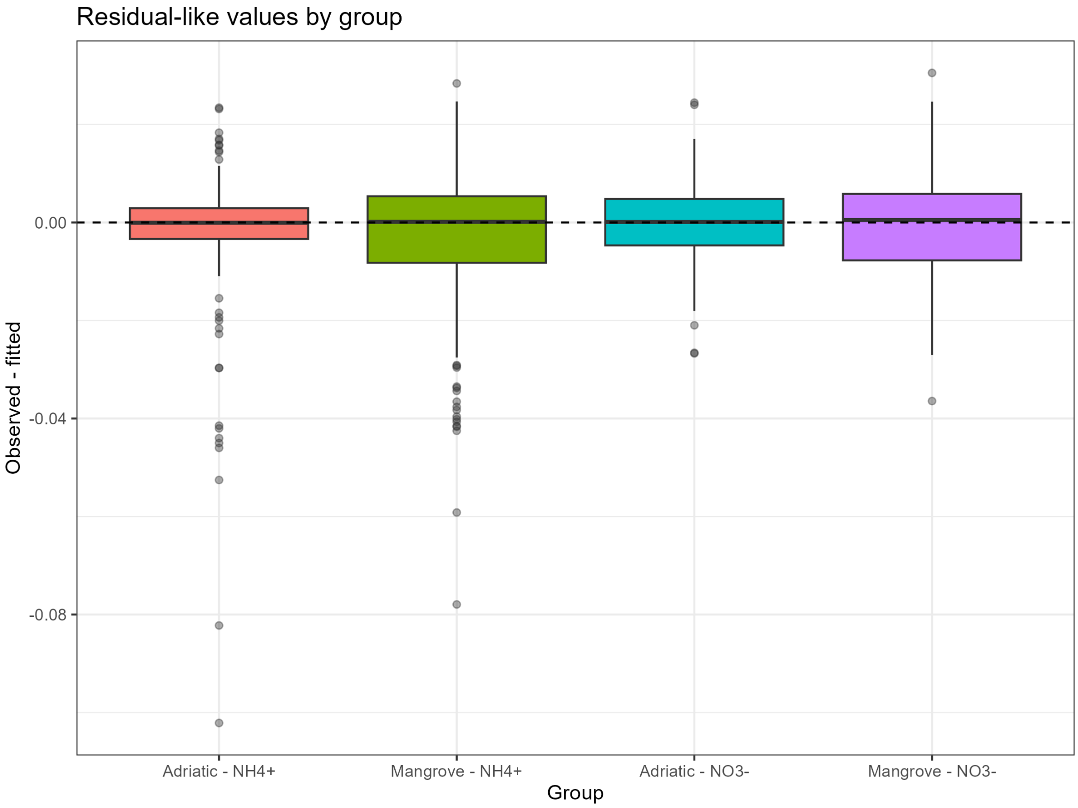

Figure S11: Residual-like values by strain × nitrogen-source group.

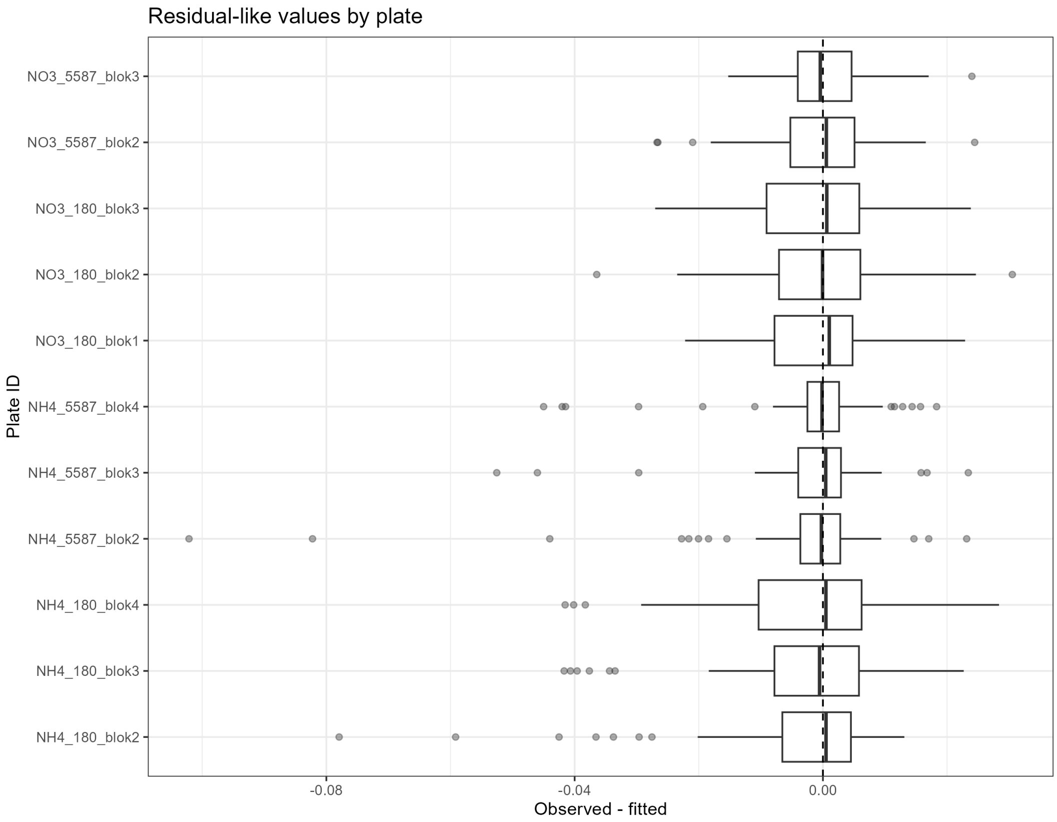

Figure S12: Residual-like values by experimental plate.

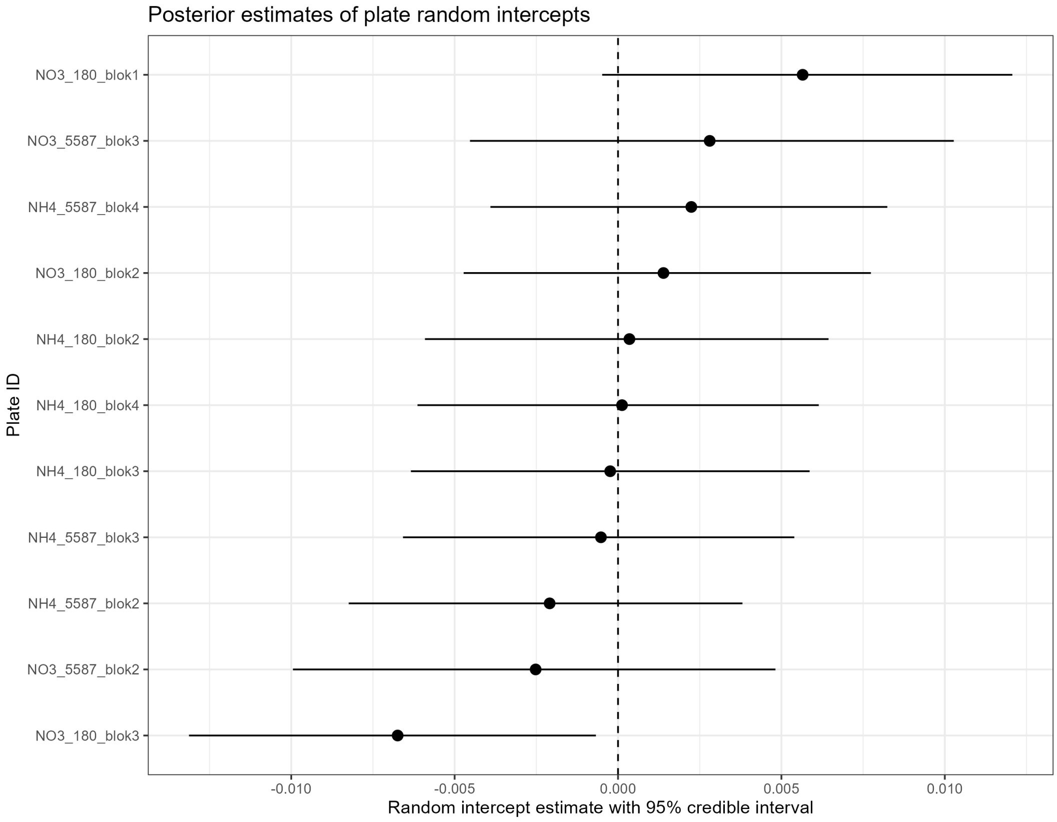

Figure S13: Posterior estimates of plate-level random intercepts.

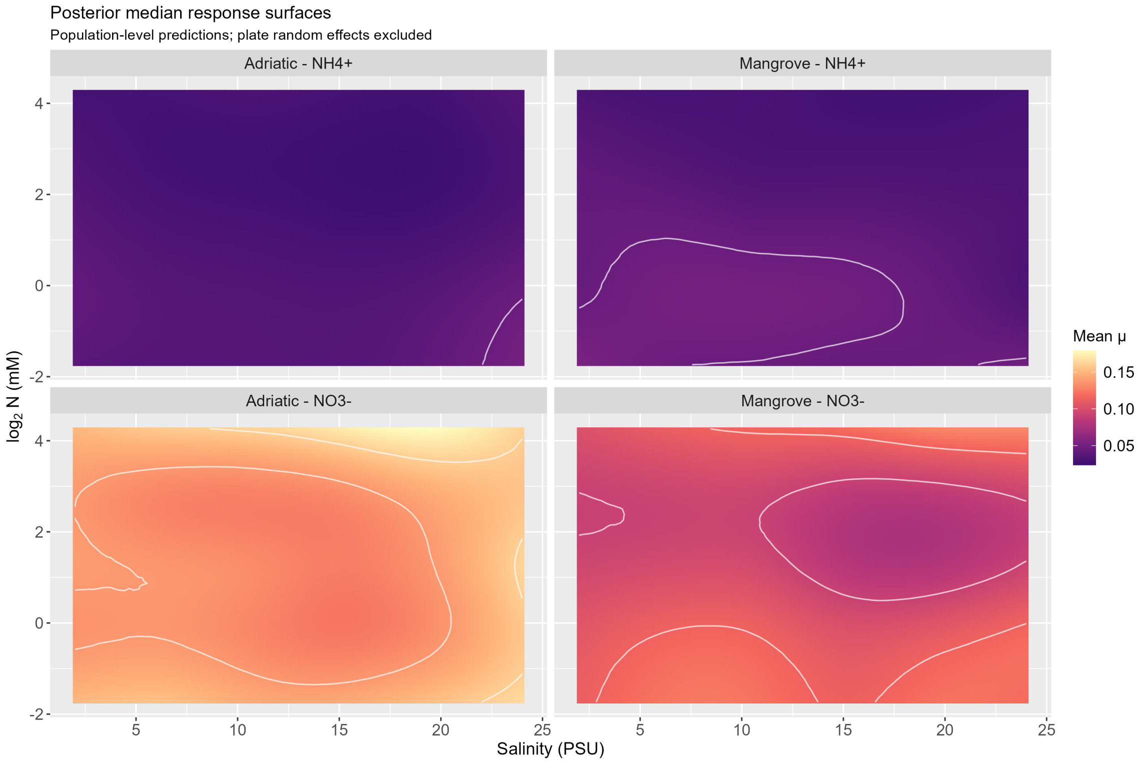

Figure S14: Posterior median population-level response surfaces, excluding plate-level random effects.

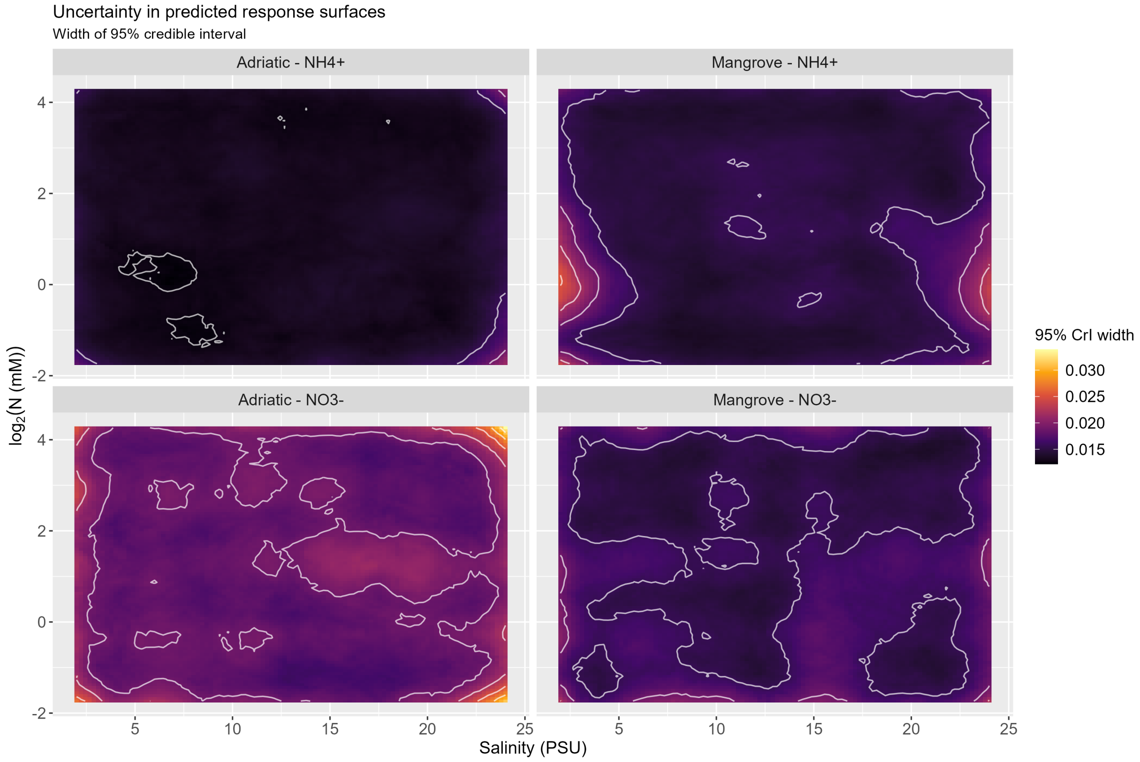

Figure S15: Prediction uncertainty surface, expressed as the width of the 95% credible interval.

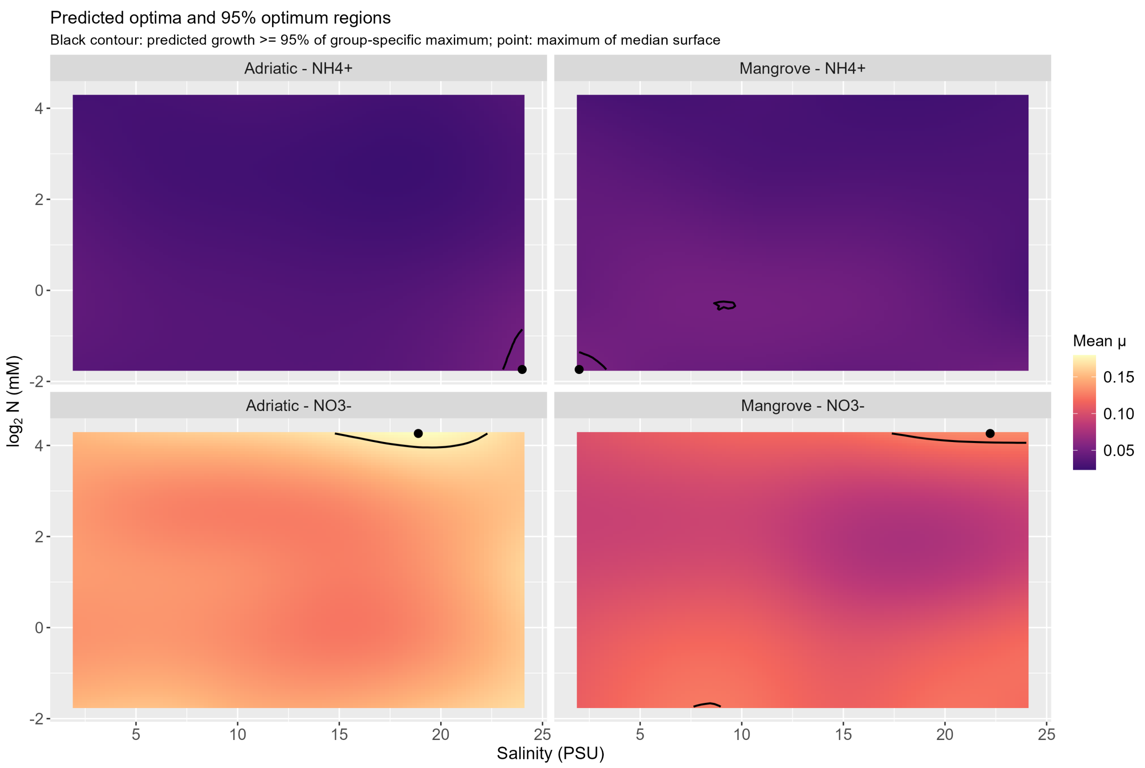

Figure S16: Predicted optima and 95% near-optimum regions. Contour indicates combinations with posterior median predicted growth at least 95% of the group-specific maximum.

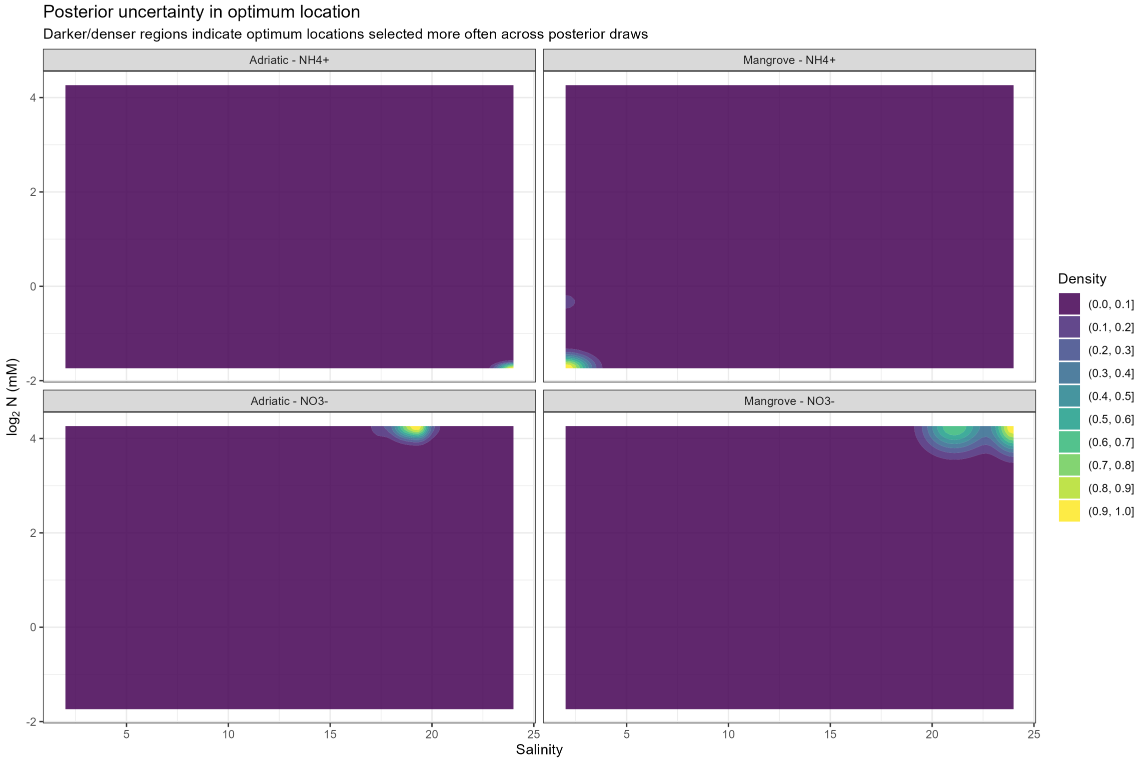

Figure S17: Posterior uncertainty in optimum location based on draw-wise maxima.

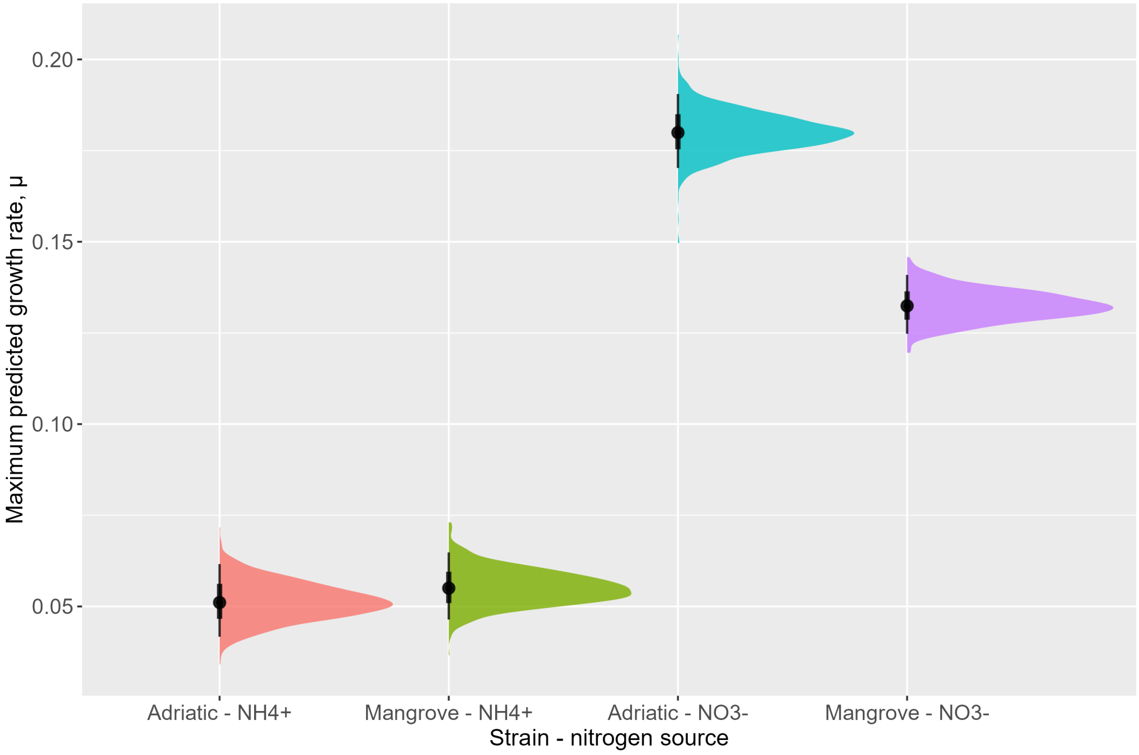

Figure S18: Posterior distributions of maximum predicted growth by group.

2 SI2: Log fold change in OD_{690}

2.1 Data structure and experimental design

The analysis included 924 observations distributed across four strain × nitrogen-source groups and 11 experimental plates. Nitrogen concentration was analysed on the $\log_2$ scale because the experimental concentration series followed a two-fold dilution design. Thus, one unit on the transformed scale corresponds to a doubling of nitrogen concentration.

Table S8: Summary of the observed log fold change in OD_{690} by strain × nitrogen-source group for log fold change in OD by group.

Group	ID	n	Plates	Mean	SD	Min	Max
Adriatic - NH4+	5587_NH4	252	3	0.5257	0.1766	-0.3996	0.9807
Mangrove - NH4+	180_NH4	252	3	0.4947	0.2243	-0.2401	0.9293
Adriatic - NO3-	5587_NO3	168	2	1.9563	0.1822	0.9992	2.3072
Mangrove - NO3-	180_NO3	252	3	1.4232	0.2099	0.7947	1.9667

2.2 Bayesian GAMM specification

Let z_i denote the log fold change in OD, $\ln\left(\frac{OD_{690,\text{final}}}{OD_{690,\text{init}}}\right)$, for observation i. The fitted model was

$$z_i \sim t_\nu(\eta_i, \sigma), \tag{S4}$$

$$\eta_i = \alpha_{g[i]} + f_{g[i]}(s_i, \log_2 N_i) + b_{p[i]}, \tag{S5}$$

$$b_p \sim \mathcal{N}(0, \sigma_{\text{plate}}), \tag{S6}$$

where $g[i]$ indexes the strain × nitrogen-source group, s_i is salinity, N_i is nitrogen concentration, and $p[i]$ indexes the experimental plate. The function $f_g(\cdot)$ is a group-specific tensor-product smooth, allowing each strain × nitrogen-source group to have a separate nonlinear salinity × nitrogen response surface.

The model was implemented in `brms` using a Student-t likelihood to ensure robustness against heavy-tailed residuals:

```
log_OD_i ~ group +
  t2(salinity, log2_N, by = group, k = c(6, 5)) +
  (1 | plate_ID)
family = student()
```

2.3 Prior distributions

The model used weakly informative priors chosen on the observed scale of log fold change in OD. Because log fold change values were substantially larger than the corresponding growth-rate responses, prior scales were widened to avoid imposing unnecessary shrinkage while still regularizing implausibly large effects. These priors allow the observed data to dominate posterior inference while improving computational stability.

Table S9: Prior distributions for the log fold change in OD Bayesian GAMM.

Model component	Prior
Intercept	Student-$t(3, 0.1, 1.0)$
Population-level coefficients	$\mathcal{N}(0, 1.0)$
Residual scale σ	Exponential(2)
Plate-level SD σ_{plate}	Exponential(2)
Student-t df ν	Gamma$(2, 0.1)$

2.4 Posterior computation and MCMC diagnostics

The model was fitted using four Markov chain Monte Carlo chains, each with 4000 iterations, including 2000 warm-up iterations. This yielded 8000 post-warmup posterior draws. Sampling was performed using Hamiltonian Monte Carlo with the No-U-Turn Sampler. Convergence and sampling quality were assessed using $\hat{R}$, effective sample size (ESS), divergent transitions, and tree-depth diagnostics.

Table S10: Summary of MCMC diagnostics for the log fold change in OD Student-t Bayesian GAMM.

Diagnostic	Value
Number of post-warmup draws	8000
Maximum $\hat{R}$	1.003
Minimum bulk ESS	1384
Minimum tail ESS	1739
Divergent transitions	0
Maximum observed tree depth	9
Student-t ν, posterior median [95% CrI]	3.44 [2.51, 5.05]

All monitored parameters had $\hat{R} \leq 1.003$. There were no divergent transitions and the sampler did not reach problematic tree-depth saturation, indicating satisfactory convergence and stable posterior sampling.

The fitted model estimated a degrees-of-freedom parameter = 3.44 (95% CrI: 2.51–5.05), indicating moderate heavy-tailed residual behavior and supporting the use of a robust Student-t likelihood over a Gaussian model.

2.5 Model validation

Model validation was based on posterior predictive checks, residual-like diagnostics, and inspection of plate-level random effects.

2.5.1 Posterior predictive checks

Posterior predictive checks compare the observed data with replicated datasets simulated from the fitted posterior predictive distribution. If the model is adequate, the observed data should look typical relative to the model-generated data.

The overall posterior predictive mean showed only a small discrepancy: the observed mean log fold change in OD was 1.022, whereas the posterior predictive median was 1.045 (95% interval: 1.026–1.064). The absolute difference was 0.023, corresponding to a small tendency to overpredict the overall mean, but not one considered biologically substantial.

The density, ECDF, mean, standard deviation, and grouped density posterior predictive checks are shown in Supplementary Figures S23–S28.

2.5.2 Residual-like checks

Although residuals are not the primary diagnostic in Bayesian posterior predictive workflows, residual-like values $z_i - \mathbb{E}(z_i \mid \text{posterior})$ were used to inspect systematic bias across fitted values, groups, and plates. These diagnostics are shown in Supplementary Figures S29–S31.

2.5.3 Plate-level variation

Plate identity was included as a random intercept to account for block-level variation. Posterior estimates were minimal and centered around zero, showing no consistent directional bias related to nitrogen source. Observed shifts represent random experimental noise rather than systematic treatment effects, confirming that block-level variation was controlled without dominating the primary biological signals.

Table S11: Posterior estimates of plate-level random intercepts for log fold change in OD. 5587: Adriatic strain, 180: Mangrove strain.

Plate ID	Estimate	95% CrI
`NH4_5587_blok2`	−0.022	[−0.111, 0.061]
`NH4_5587_blok3`	−0.002	[−0.089, 0.083]
`NH4_5587_blok4`	0.015	[−0.073, 0.099]
`NH4_180_blok2`	0.029	[−0.058, 0.119]
`NH4_180_blok3`	−0.006	[−0.095, 0.083]
`NH4_180_blok4`	−0.022	[−0.111, 0.068]
`NO3_5587_blok2`	−0.034	[−0.140, 0.074]
`NO3_5587_blok3`	0.043	[−0.060, 0.150]
`NO3_180_blok1`	0.081	[−0.005, 0.171]
`NO3_180_blok2`	0.016	[−0.072, 0.107]
`NO3_180_blok3`	−0.092	[−0.182, −0.006]

2.6 Robustness and sensitivity analyses

The following robustness checks were implemented to ensure the validity of the log fold change in OD analysis and were derived from the final model outputs.

2.6.1 Sensitivity to the Gaussian residual assumption

The final model used a Student-t residual distribution because initial diagnostics indicated non-normality and heavy-tailed residuals in the observations. The fitted model estimated a degrees-of-freedom parameter of $\nu = 3.44$ (95% CrI: 2.51–5.05). This relatively low value indicates significant residual heavy-tailedness, quantitatively supporting the use of a robust Student-t likelihood over a standard Gaussian model.

2.6.2 Sensitivity to plate-level structure

The model included 11 plate-level random intercepts to account for block-level experimental variation. The posterior standard deviation for these effects was small, indicating that plate effects were accounted for but were not the primary driver of the observed variation. Furthermore, as seen in the residuals by plate, the heavy tails in the data appeared to reflect individual outlying observations rather than systematic shifts across entire experimental blocks.

2.6.3 Sensitivity of optimum inference

To ensure that inferred optima were not overly sensitive to the specific values at a single grid point, three complementary summaries were utilized:

1. The maximum of the posterior median response surface, providing a single point estimate;
2. The 95% near-optimum region, defined as all salinity and nitrogen grid points with a posterior median log fold change in OD of at least 95% of the group-specific maximum;
3. The posterior distribution of optimum locations, obtained by identifying the maximum grid point separately for each individual posterior draw.

This approach avoids overinterpreting a single grid point and directly accounts for uncertainty in the location of the optima. For the Mangrove nitrate group, the posterior optimum location was particularly broad, indicating a relatively flat near-optimal surface and justifying the interpretation of responses in terms of broader near-optimum regions rather than a single precise point.

2.7 Predicted response surfaces and optimum regions

Population-level response surfaces were predicted with plate random effects excluded, so that the surfaces represent expected group-level responses rather than any specific plate. The posterior median response surfaces and uncertainty surfaces are shown in Supplementary Figures S32 and S33.

Table S12: Point optima and 95% near-optimum regions for predicted log fold change in OD. 5587: Adriatic strain, 180: Mangrove strain.

Group	Opt. PSU	Opt. N	Max. pred [95% CrI]	95% salinity range	95% N range	Grid points
5587_NH4	24.0	0.4	0.775 [0.648, 0.908]	[22.4, 24.0]	[0.3, 0.86]	162
180_NH4	24.0	0.3	0.725 [0.595, 0.852]	[2.0, 24.0]	[0.3, 1.2]	1340
5587_NO3	24.0	2.5	2.225 [2.082, 2.366]	[2.0, 24.0]	[0.3, 19.2]	486
180_NO3	7.8	0.3	1.656 [1.546, 1.782]	[3.6, 24.0]	[0.3, 19.2]	1122

The strongest log fold change in OD response occurred in nitrate media (TableS12. The highest maximum predicted log fold change was estimated for Adriatic strain in nitrate (`5587_NO3`), with a posterior median maximum of 2.225. The nitrate response of Mangrove strain (`180_NO3`) also exceeded both ammonium responses, with a maximum predicted value of 1.656. In contrast, both ammonium groups had substantially lower maximum predicted responses, with maxima below 0.8 and optima occurring at low ammonium concentrations.

Table S13: Values are posterior medians with 95% credible intervals obtained from draw-wise maximum locations. 5587: Adriatic strain, 180: Mangrove strain.

Group	Salinity (PSU)	N concentration (mM)	Log fold change in OD
5587_NH4	24.0 [2.0, 24.0]	0.3 [0.3, 0.79]	0.792 [0.678, 0.937]
180_NH4	8.9 [2.0, 24.0]	0.6 [0.3, 0.97]	0.770 [0.661, 0.882]
5587_NO3	24.0 [2.0, 24.0]	1.6 [0.3, 5.36]	2.257 [2.131, 2.399]
180_NO3	20.7 [6.2, 24.0]	0.7 [0.3, 19.2]	1.688 [1.595, 1.810]

For the Mangrove strain (`180_NH4` and `180_NO3`), there is substantial uncertainty in optimum location, with mismatch of summaries due to flat and multimodal response surface. Methodological differences between the median surface peak (Table S12) and the distribution of draw-wise peaks (Table S13) result in divergent point optima for these groups. Biological interpretations should therefore prioritize broad near-optimum regions over specific coordinates.

2.8 Pairwise comparisons of maximum predicted responses

Pairwise comparisons were computed from posterior draws of the maximum predicted log fold change in OD for each group. These comparisons summarize differences in group-specific maxima and should not be interpreted as pointwise comparisons across the entire response surface.

Table S14: Pairwise posterior differences in maximum predicted log fold change in OD. Positive values indicate that the first group has a larger maximum than the second group.

Contrast	Median difference	95% CrI	Pr(diff > 0)
5587_NH4 - 180_NH4	0.024	[-0.134, 0.201]	0.604
5587_NH4 - 5587_NO3	-1.463	[-1.645, -1.274]	0.000
5587_NH4 - 180_NO3	-0.899	[-1.062, -0.731]	0.000
180_NH4 - 5587_NO3	-1.491	[-1.666, -1.320]	0.000
180_NH4 - 180_NO3	-0.920	[-1.076, -0.764]	0.000
5587_NO3 - 180_NO3	0.565	[0.404, 0.732]	1.000

The posterior comparisons showed a clear ranking of maximum predicted log fold change in OD:

$$\texttt{5587\_NO3} > \texttt{180\_NO3} \gg \texttt{5587\_NH4} \approx \texttt{180\_NH4}.$$

Both nitrate groups exhibited substantially larger maximum predicted log fold changes than either ammonium group. The posterior probability that the Adriatic nitrate strain (`5587_NO3`) exceeded the Mangrove nitrate strain (`180_NO3`) was effectively 1.00, with a median difference of 0.565. In contrast, the two ammonium groups showed little evidence of a meaningful difference, with a median difference of only 0.024 and a 95% credible interval spanning zero.

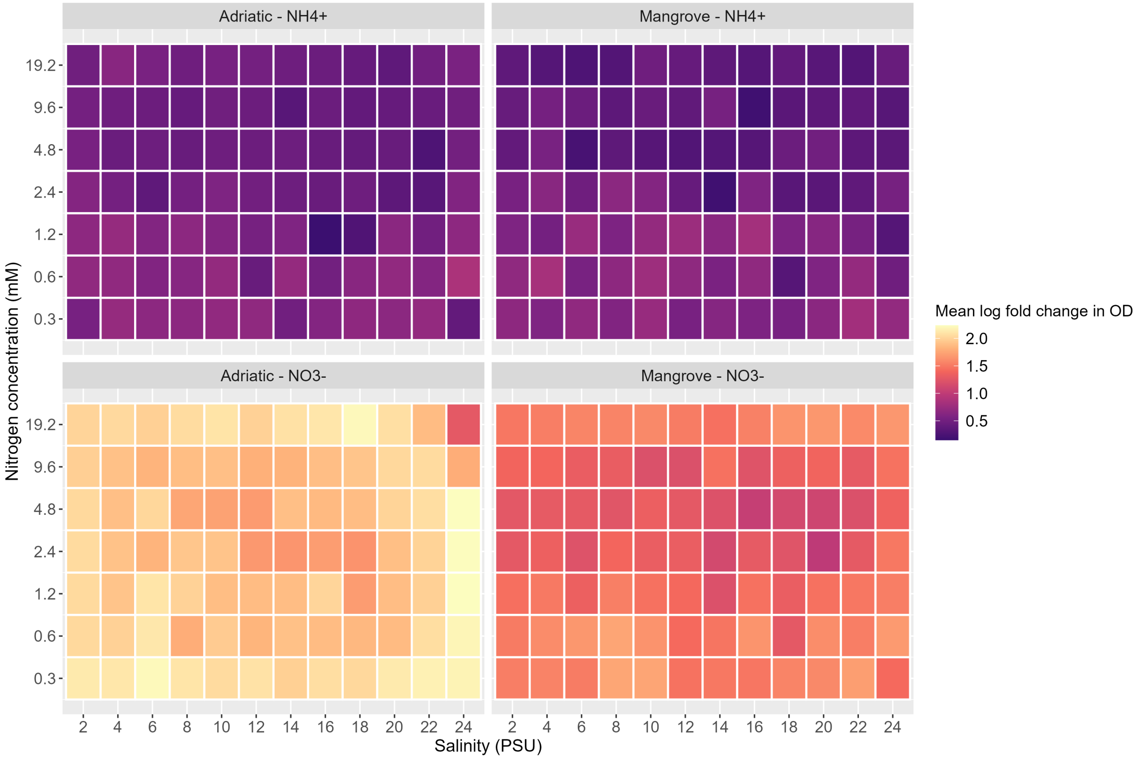

Figure S19: Observed mean log fold change in OD surface by group. This plot summarizes raw means before model fitting and provides a visual check that the modelled response surfaces reflect the empirical structure of the data.

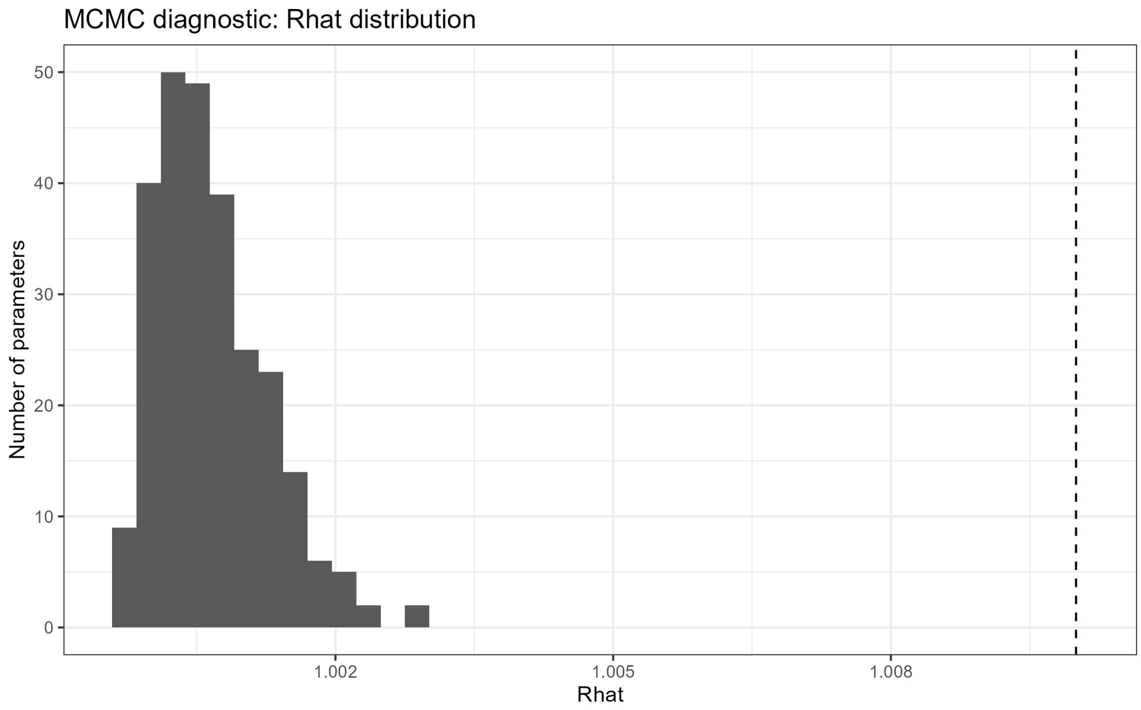

Figure S20: Distribution of $\hat{R}$ values across monitored parameters for the log fold change in OD model. Values close to 1 indicate agreement among MCMC chains.

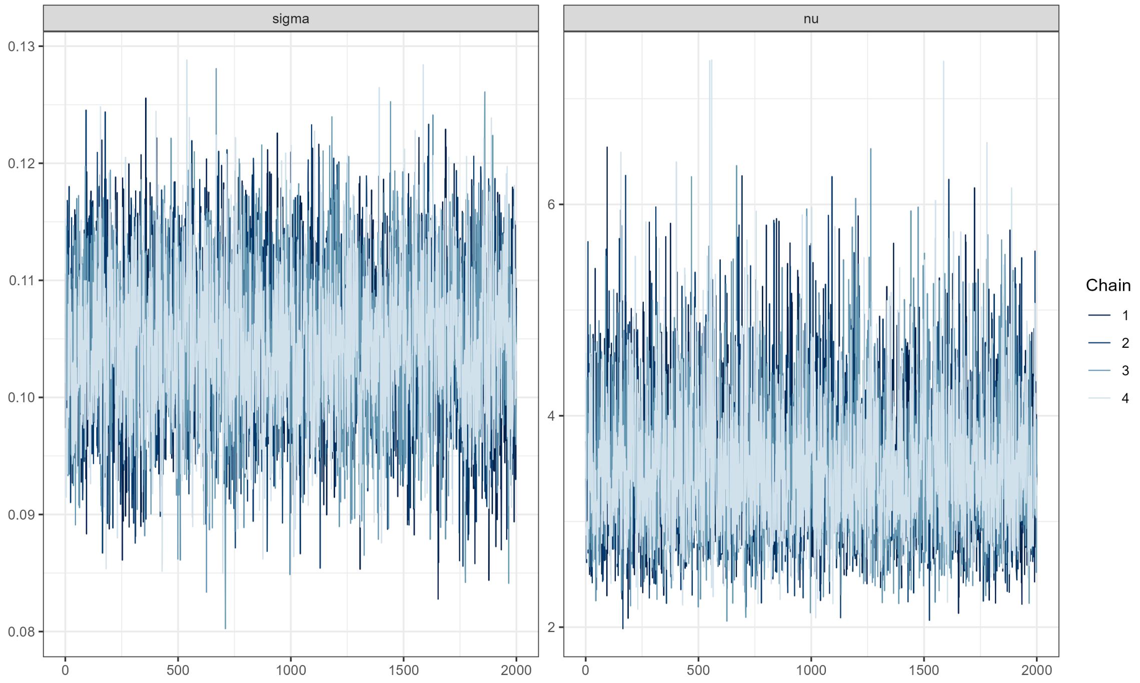

Figure S21: Trace plots for residual scale σ and Student-t degrees-of-freedom parameter ν in the log fold change in OD model. Well-mixed chains without drift support stable posterior sampling.

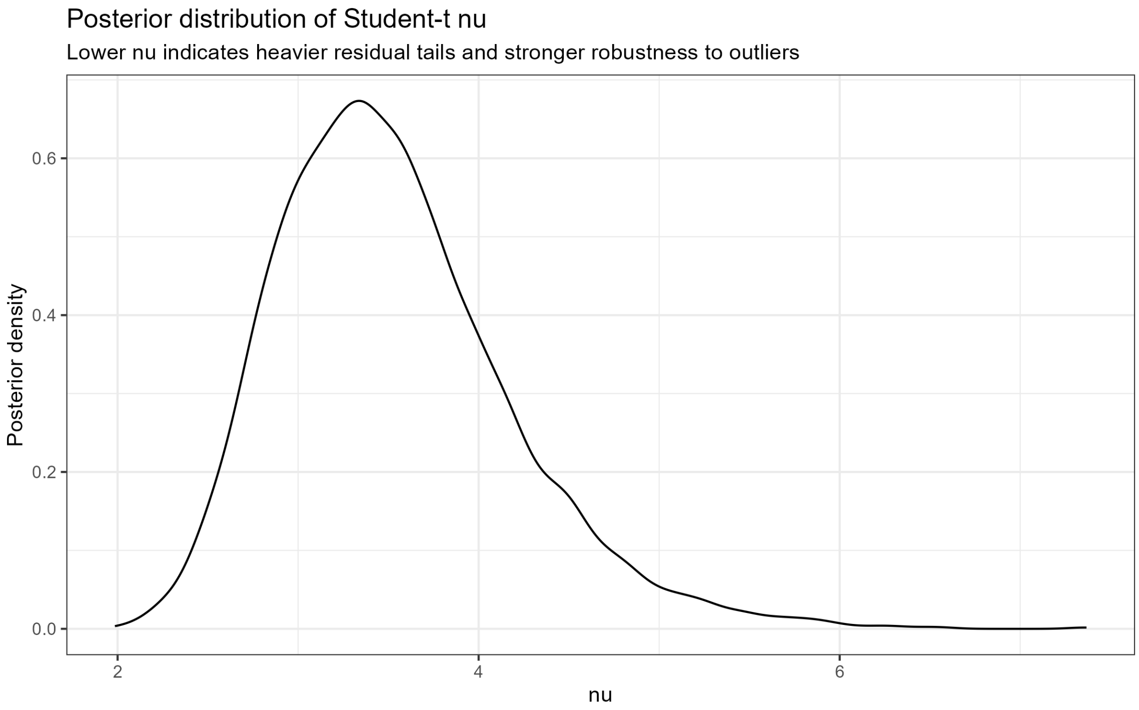

Figure S22: Posterior distribution of the Student-t degrees-of-freedom parameter for the log fold change in OD model. Low values indicate heavy-tailed residual variation.

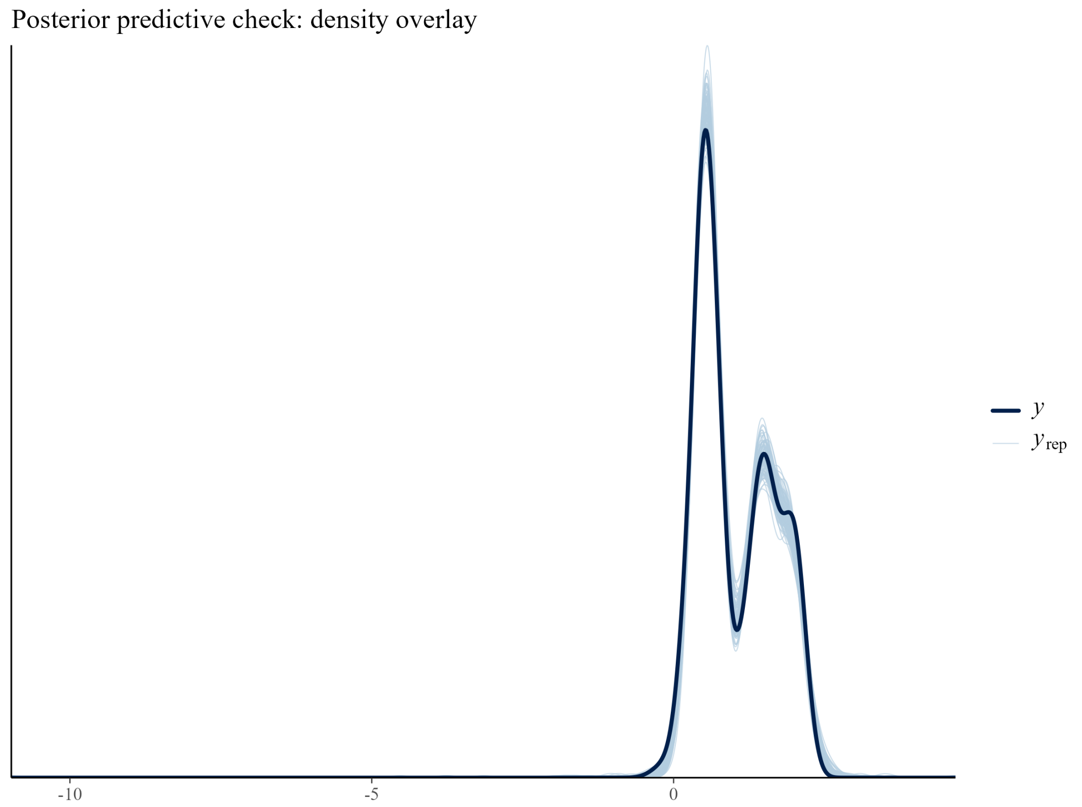

Figure S23: Posterior predictive density overlay for the marginal distribution of log fold change in OD.

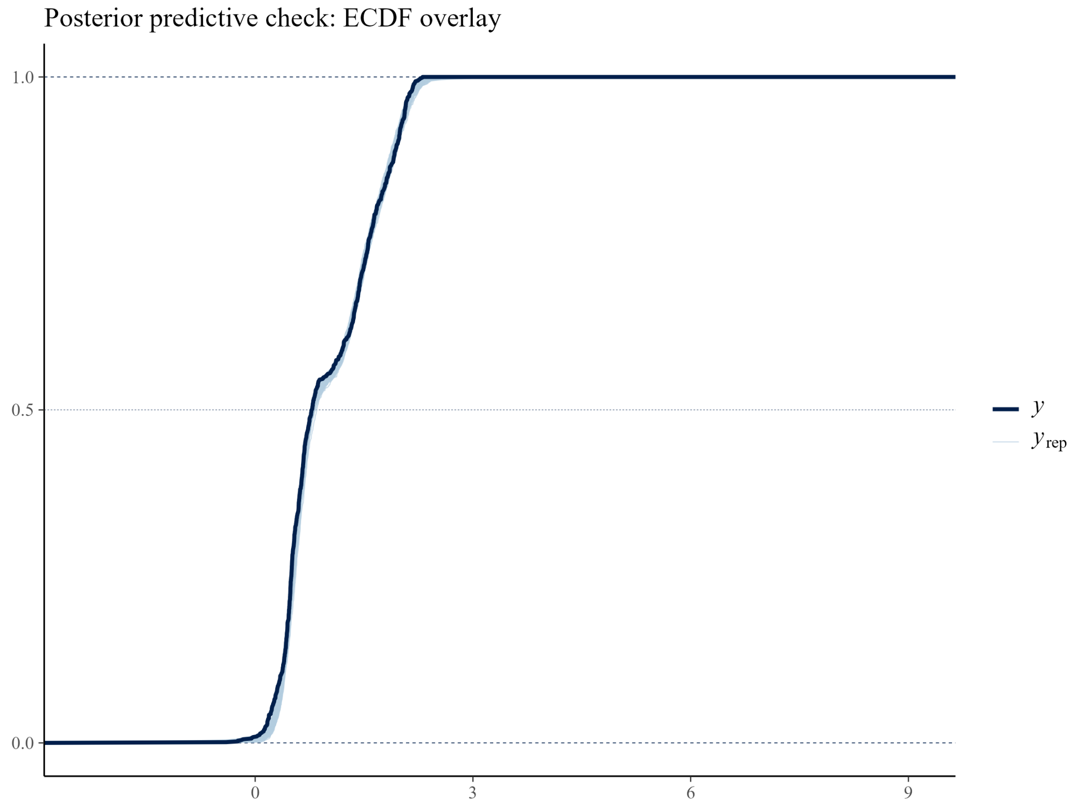

Figure S24: Posterior predictive ECDF overlay for log fold change in OD.

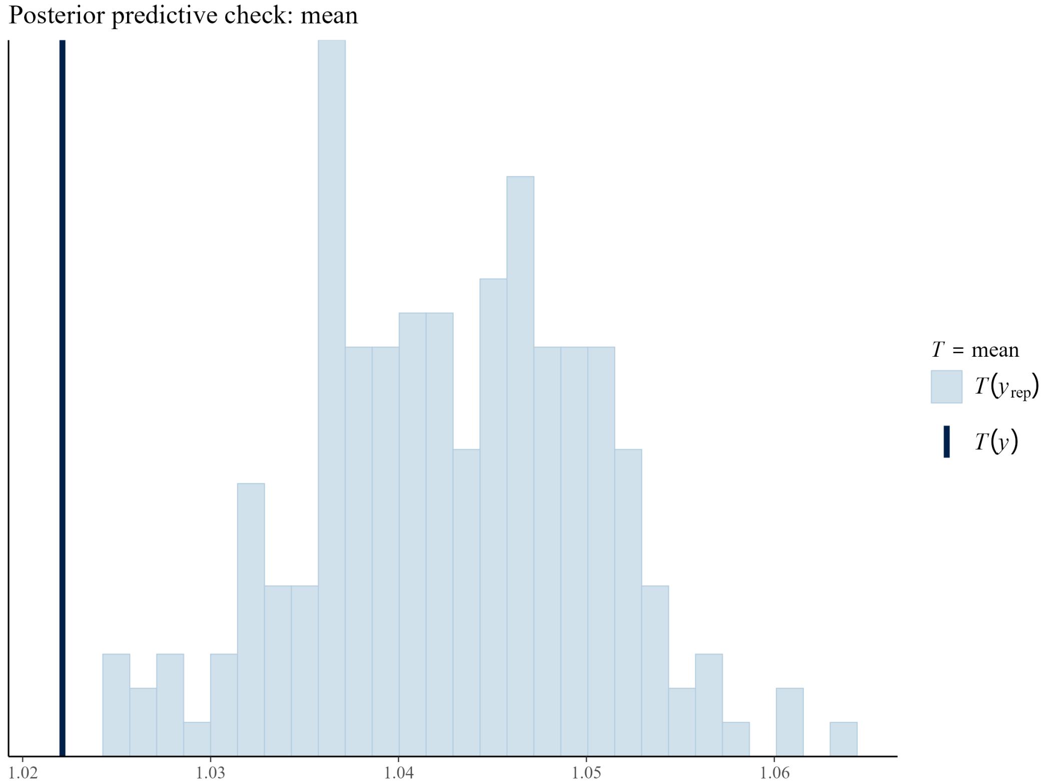

Figure S25: Posterior predictive check for the overall mean of log fold change in OD.

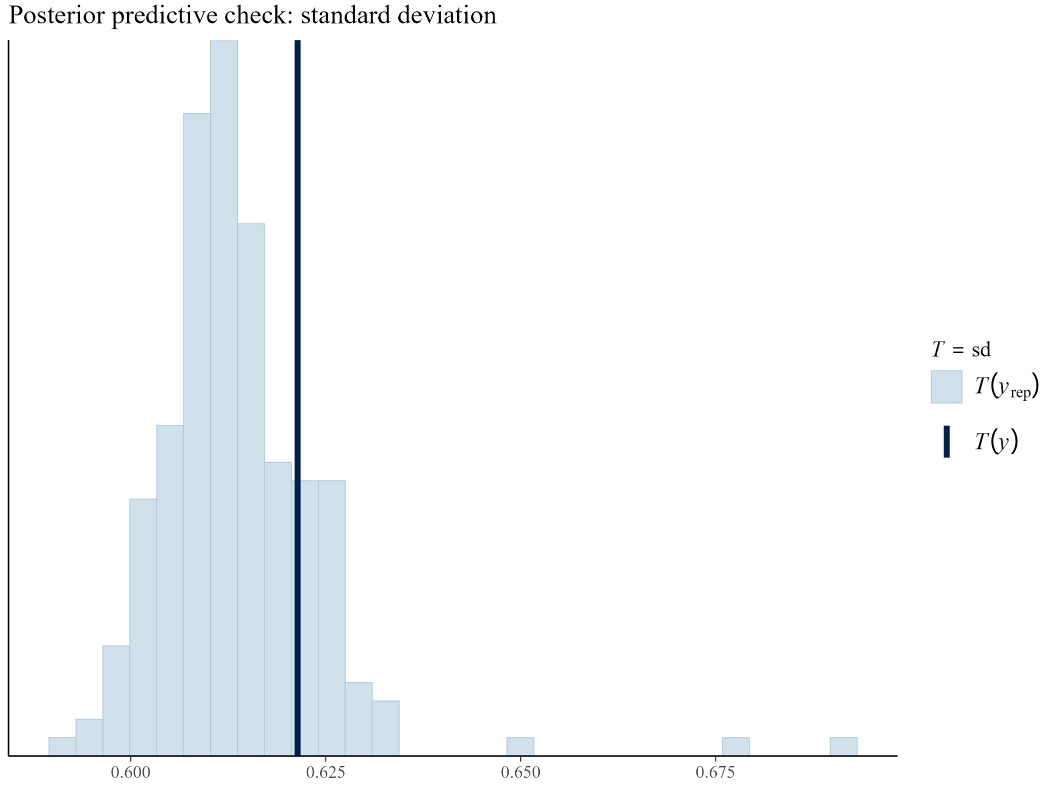

Figure S26: Posterior predictive check for the overall standard deviation of log fold change in OD.

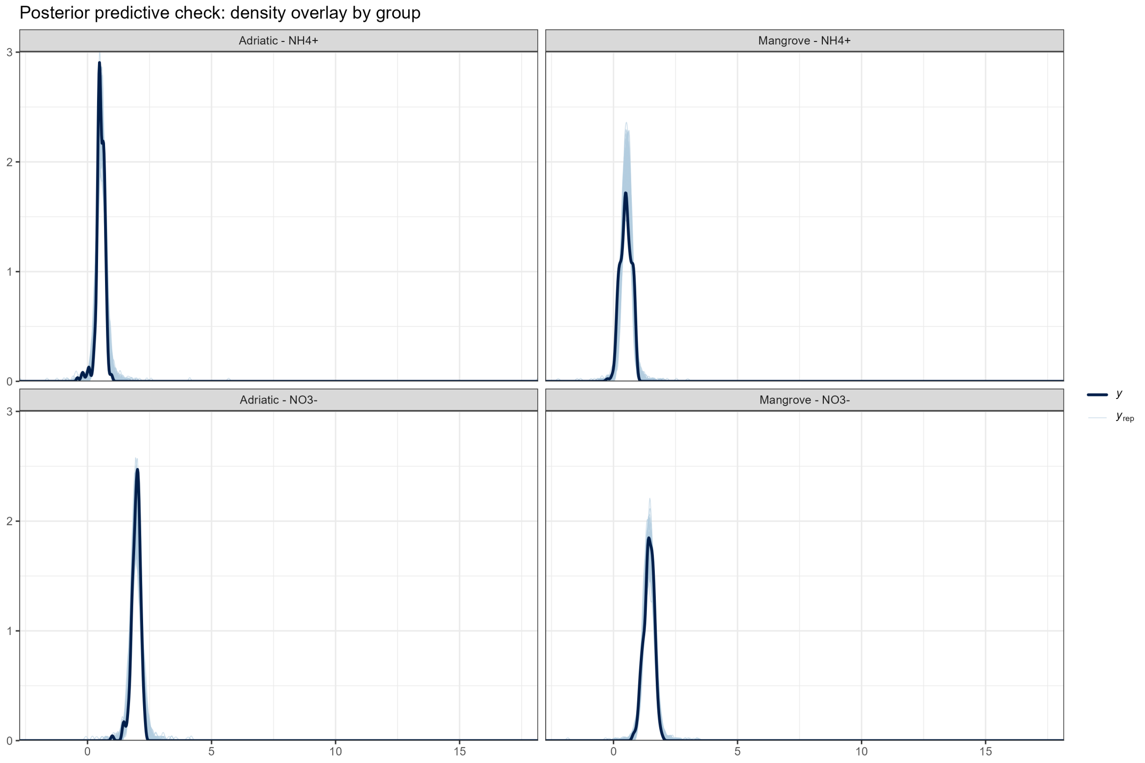

Figure S27: Group-specific posterior predictive density overlays for log fold change in OD.

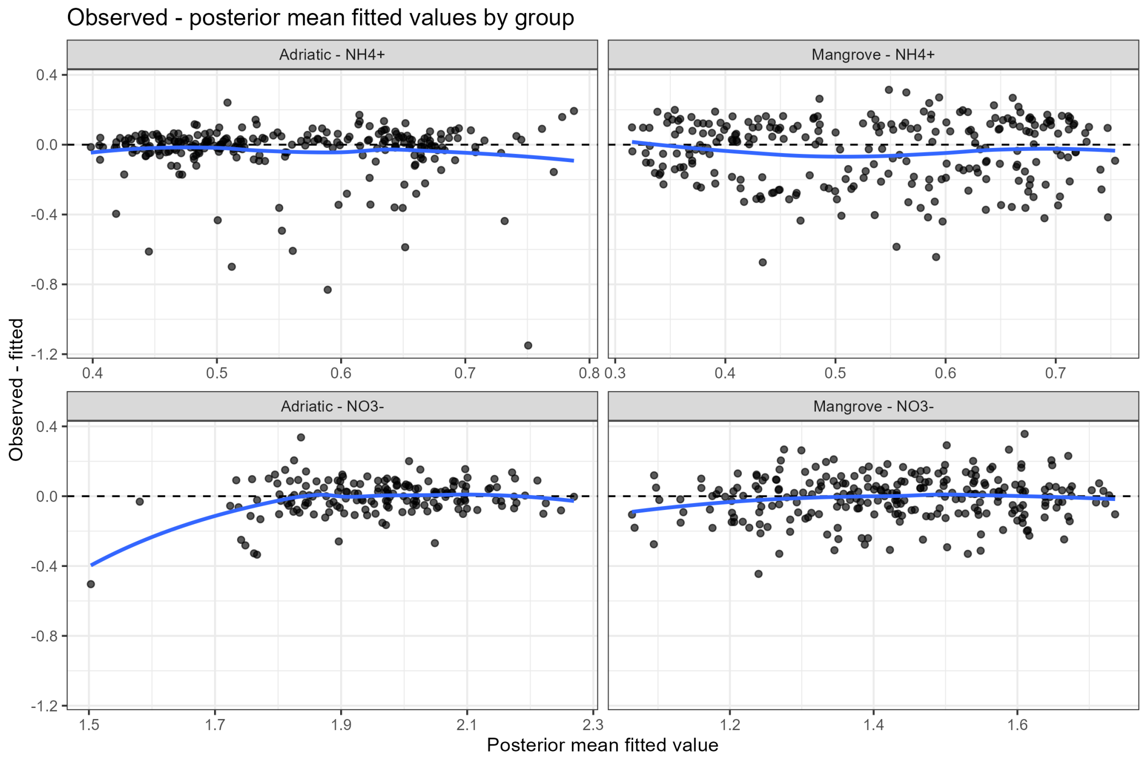

Figure S28: Residual-like values plotted against posterior mean fitted values for log fold change in OD, separately by group.

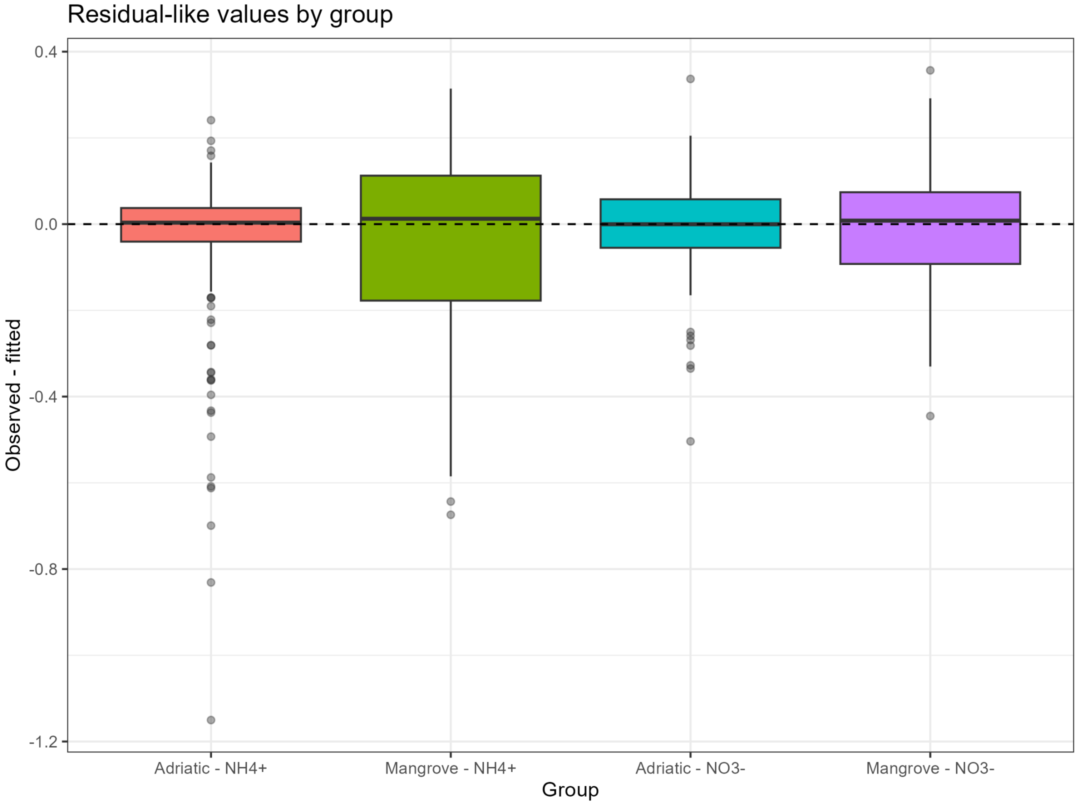

Figure S29: Residual-like values by strain × nitrogen-source group for log fold change in OD.

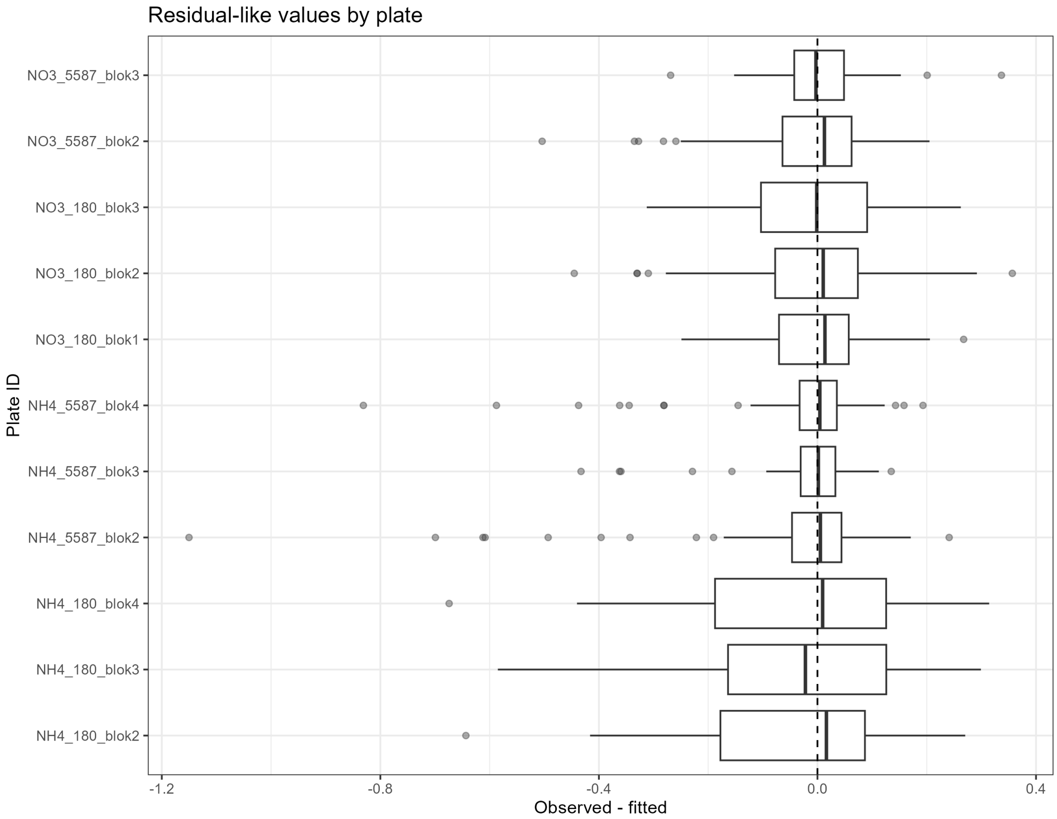

Figure S30: Residual-like values by experimental plate for log fold change in OD.

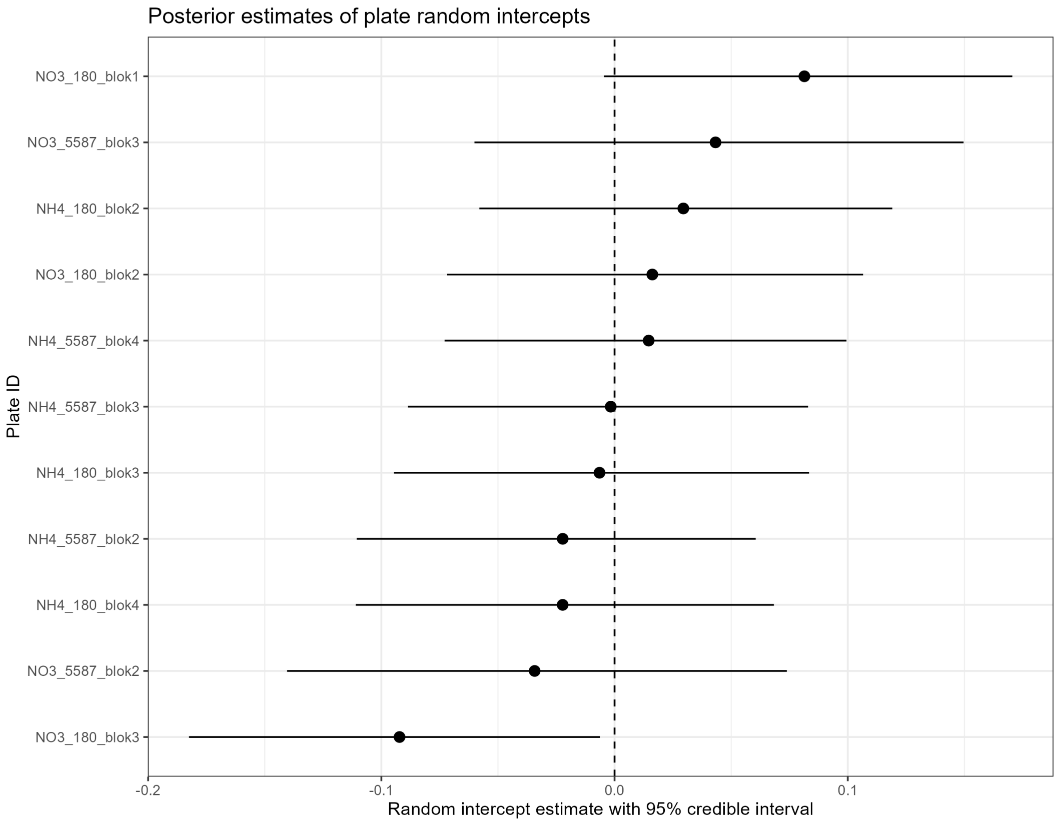

Figure S31: Posterior estimates of plate-level random intercepts for log fold change in OD.

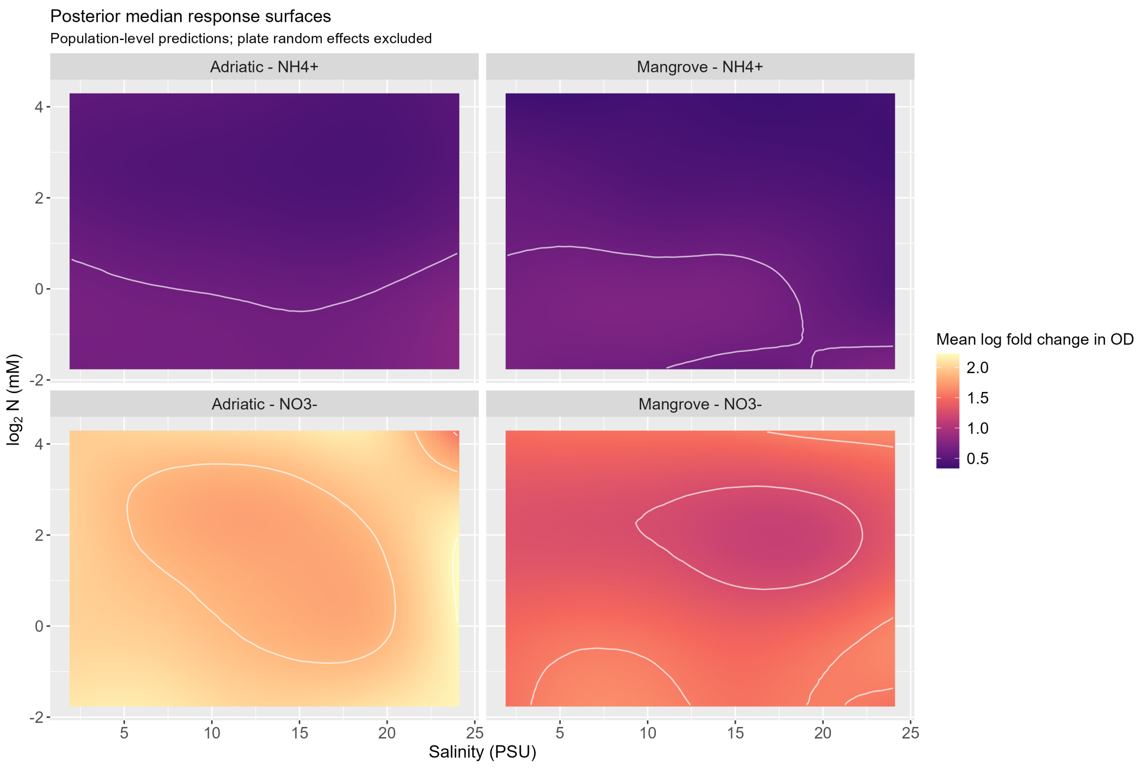

Figure S32: Posterior median population-level response surfaces for log fold change in OD, excluding plate-level random effects.

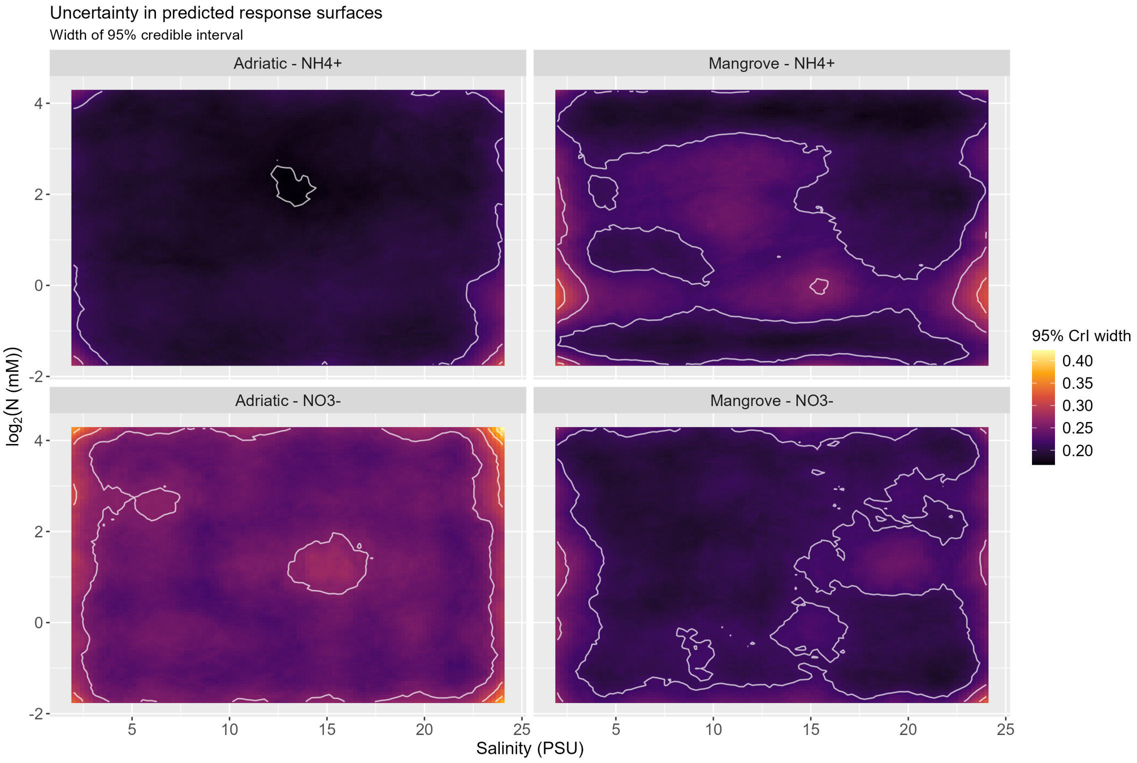

Figure S33: Prediction uncertainty surface (95% credible interval width) for log fold change in OD.

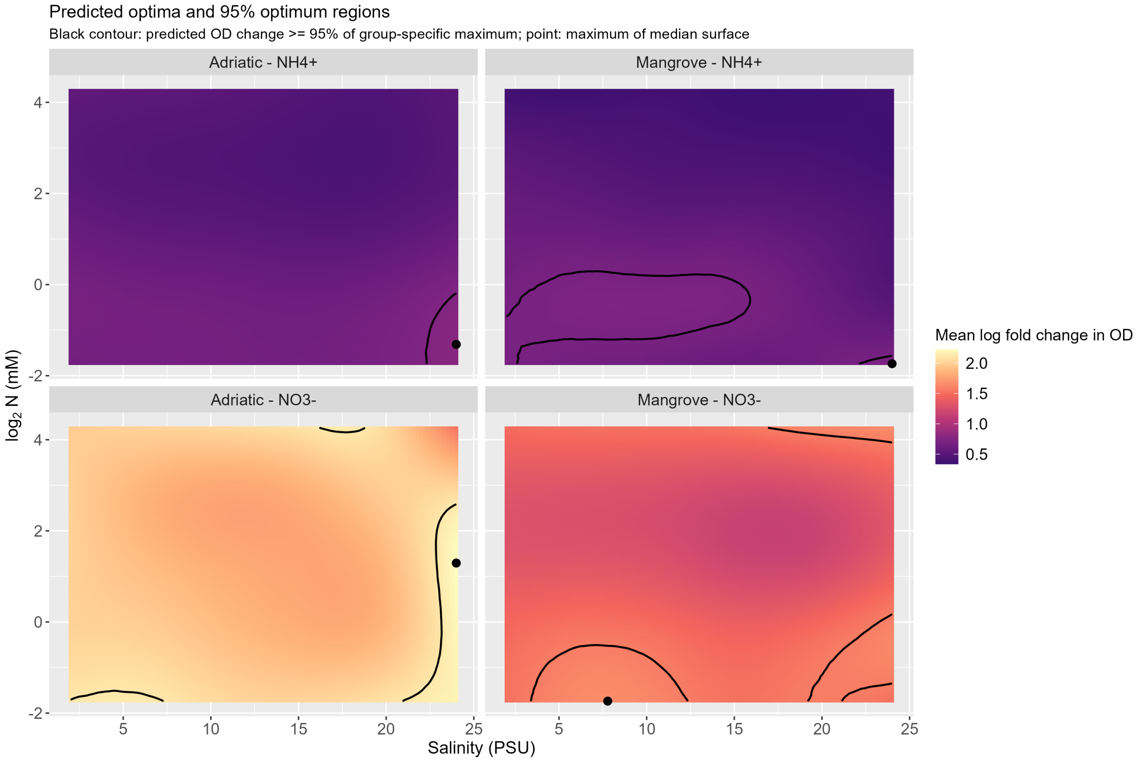

Figure S34: Predicted optima and 95% near-optimum regions for log fold change in OD. Contours indicate combinations with posterior median response at least 95% of the group-specific maximum.

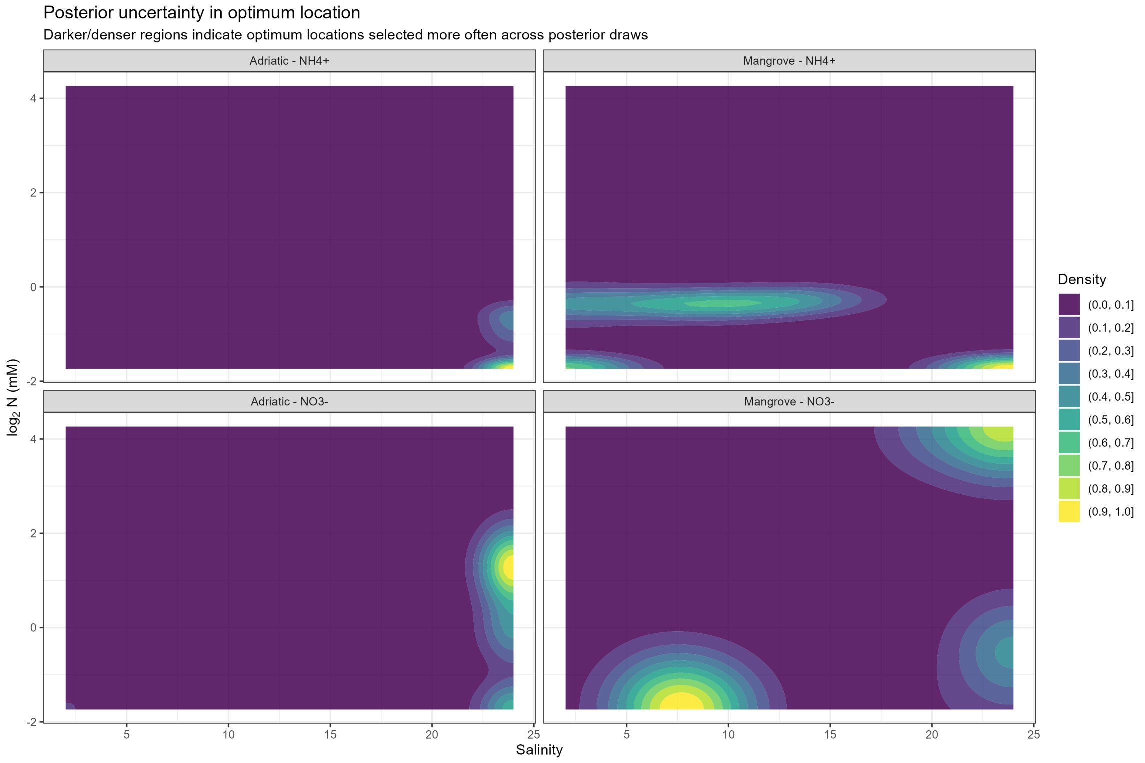

Figure S35: Posterior uncertainty in optimum location based on draw-wise maxima for log fold change in OD.

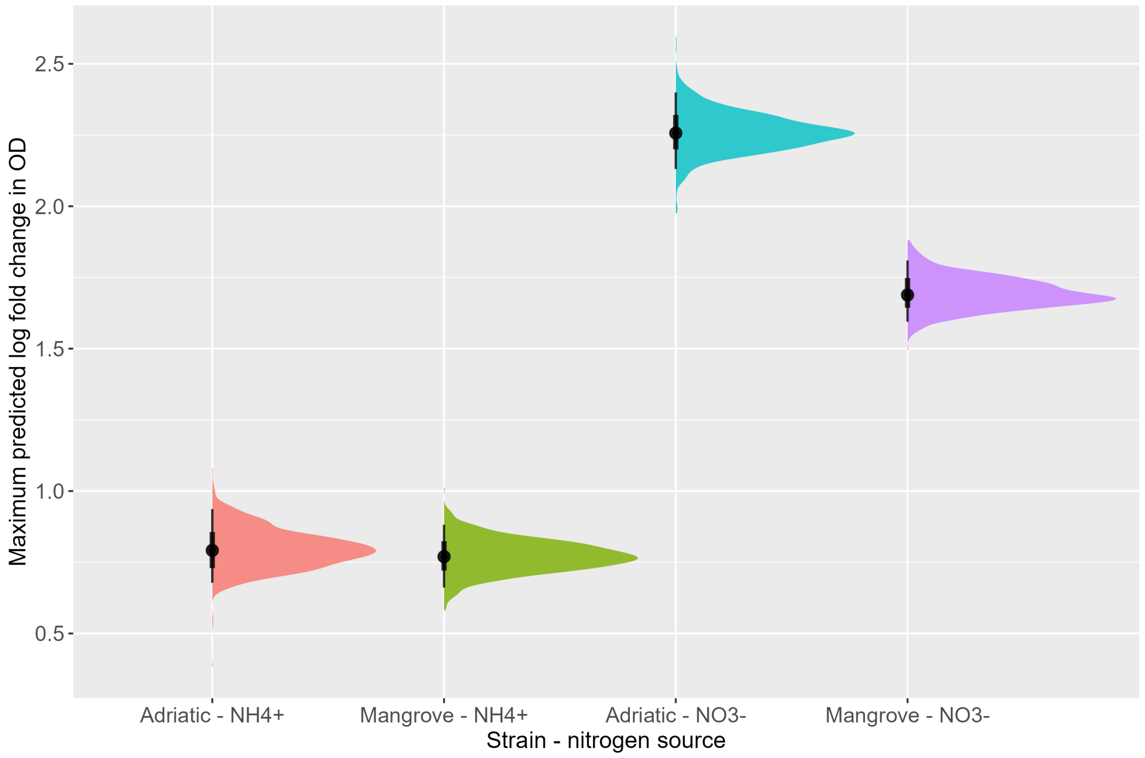

Figure S36: Posterior distributions of the maximum predicted log fold change in OD for each experimental group.

References

[1] Bürkner, P.-C. (2017). *brms: An R Package for Bayesian Multilevel Models Using Stan.* Journal of Statistical Software, 80(1), 1–28.

[2] Hoffman, M. D. and Gelman, A. (2014). *The No-U-Turn Sampler: Adaptively Setting Path Lengths in Hamiltonian Monte Carlo.* Journal of Machine Learning Research, 15, 1593–1623.

[3] Gelman, A., Carlin, J. B., Stern, H. S., Dunson, D. B., Vehtari, A., and Rubin, D. B. (2013). *Bayesian Data Analysis*, 3rd ed. CRC Press.
